# A comparison of calcium hydroxide/iodoform paste and zinc oxide eugenol as root filling materials for pulpectomy in primary teeth: A systematic review and meta‐analysis

**DOI:** 10.1002/cre2.173

**Published:** 2019-03-04

**Authors:** Rahaf S. Najjar, Najlaa M. Alamoudi, Azza A. El‐Housseiny, Amani A. Al Tuwirqi, Heba J. Sabbagh

**Affiliations:** ^1^ Pediatric Dentistry Department, Faculty of Dentistry King Abdulaziz University Jeddah Saudi Arabia; ^2^ Pediatric Dentistry Department, Faculty of Dentistry Alexandria University Alexandria Egypt

**Keywords:** Ca(OH)_2_/iodoform, meta‐analysis, primary teeth, pulpectomy, zinc oxide eugenol (ZOE)

## Abstract

Zinc oxide eugenol (ZOE) has traditionally been used as a root filling material in primary teeth pulpectomy. Calcium hydroxide and iodoform (Ca(OH)_2_/iodoform) may have advantages as a root canal filling material to evaluate treatment success of Ca(OH)_2_/iodoform pulpectomy in primary teeth compared with ZOE based on clinical and radiographical criteria. All human clinical studies reporting clinical and radiographical outcomes of Ca(OH)_2/_iodoform compared with ZOE in primary teeth pulpectomy were identified in digital bibliographic databases. Two authors independently selected studies and extracted relevant study characteristics. Success of treatment was based on an accomplishment of specific clinical and radiographical criteria. Meta‐analyses were performed to appraise study heterogeneity and aggregated statistics. Out of 5,000 articles identified in initial search, 15 articles met all inclusion criteria, while 10 were included in the meta‐analyses. At 6‐ and 12‐month follow‐up, there were no statistically significant differences in the clinical and radiographical success rates of Ca(OH)_2_/iodoform and ZOE. However, ZOE was shown to have statistically significant higher success rates at ≥18‐month follow‐up. On the basis of the findings of this systematic review, we recommend that Ca(OH)_2_/iodoform be utilized for pulpectomy in primary teeth nearing exfoliation; conversely, ZOE should be utilized when exfoliation is not expected to occur soon. Future randomized control clinical trials with a long‐term follow‐up are needed before a reliable conclusion can be drawn as to the best pulpectomy material. The success of pulpectomy in primary teeth depends on selecting the ideal root canal filling material. It is challenging to select the appropriate filling materials for primary teeth. ZOE or ZOE/iodoform combined with Ca(OH)_2_ appears to be the materials of choice if primary teeth are not nearing exfoliation. More high‐quality randomized control clinical trials with a long‐term follow‐up period are needed before a reliable conclusion can be drawn as to the best pulpectomy material in primary teeth (systematic review registration number: CRD42016037563).

**Why this paper is important:**

- The success of pulpectomy in primary teeth depends on selecting the ideal root canal filling material. It is challenging to select the appropriate filling materials for primary teeth.
- Zinc oxide eugenol or zinc oxide eugenol/iodoform combined with Ca(OH)_2_ appears to be the materials of choice if primary teeth are not nearing exfoliation.
- More high‐quality randomized control clinical trials with a long‐term follow‐up period are needed before a reliable conclusion can be drawn as to the best pulpectomy material in primary teeth.

## 1 INTRODUCTION

Dental caries is a worldwide public health problem commonly affecting children in their early childhood with a negative impact on childrens' oral as well as general health (Finucane, 2012). When caries reaches the pulp, one or more of the following signs and symptoms may occur: spontaneous pain especially at night, pain on biting, intraoral swelling, or intraoral sinus tract formation (Rodd, Waterhouse, Fuks, Fayle, & Moffat, 2006). Two alternative treatments in such cases are tooth extraction or root canal treatment (pulpectomy; Moskovitz, Sammara, & Holan, 2005). Root canal treatment was introduced as early as 1932 as a way to save primary teeth that otherwise would have been extracted (Kubota, Golden, & Penugonda, 1992).

The criteria of an ideal root canal filling material in primary teeth are as follows: being antibacterial, resorbs at the same rate as the roots and not causing harms to the periapical area, and the development of the succedaneous tooth. Also, it should fill the canal easily, adhere to the wall of the canal, resorb if extruded beyond the apex, show radio‐opaque appearance in the radiograph, and do not cause discoloration to the tooth (Garcia‐Godoy, 1987; Rifkin, 1980).

Zinc oxide eugenol (ZOE) has been the conventional root canal filling material used for primary teeth pulpectomy since 1930. ZOE has several disadvantages: low resorption rate (Erausquin & Muruzabal, 1967), causing irritation to the periapical area (Spedding, 1985), necrosis to bone and cementum (Hendry, Jeansonne, Dummett, & Burrell, 1982), and deflection of the permanent tooth bud (Coll & Sadrian, 1996). Studies report that the success rate of ZOE alone or with fixative medications as formocresol or iodoform ranges from 65% to 86% (Coll, Josell, & Casper, 1985; Holan & Fuks, 1993).

In 1920, calcium hydroxide (Ca(OH)_2_), a silicone oil‐based paste, was introduced by Hermann and has been widely used. Iodoform has been added to Ca(OH)_2_ due to its antibacterial effect (Estrela, Estrela, Hollanda, Decurcio, & Pécora, 2006), healing properties, and ability to be resorbed when in excess (Nurko, Ranly, Garcia‐Godoy, & Lakshmyya, 2000). The reported success rate for the combined Ca(OH)_2_/iodoform paste ranges from 84% to 100% (Reddy & Fernandes, 1996). Additional benefits of iodoform include its radiopacity, the ease with which it can be introduced and removed from the canal, negative effect on the succedaneous tooth, and its ability to be resorbed within 8 weeks once it has been extruded beyond the apex (Nurko & Garcia‐Godoy, 1999). The main disadvantage of Ca(OH)_2_/iodoform paste is a potential risk of intracanal resorption (Nurko et al., 2000).

There are no comprehensive studies that examine the clinical and radiographical outcomes of Ca(OH)_2_/iodoform as a pulpectomy material in primary teeth. Therefore, the aim of this systematic review (SR) and meta‐analysis was to evaluate treatment success of Ca(OH)_2_/iodoform pulpectomy in primary teeth compared with ZOE based on clinical and radiographical criteria.

## 2 MATERIALS AND METHODS

This study was written according to the Preferred Reporting Items for Systematic Reviews and Meta‐Analysis statement (Liberati et al., 2009). It was registered in the International Prospective Register of Systematic Reviews with registration number CRD42016037563.

### 2.1 Selection criteria

Studies reporting clinical and radiographical outcomes of Ca(OH)_2_/iodoform compared with ZOE pulpectomy in primary teeth were considered as eligible. The inclusion criteria were randomized and non‐randomized clinical trials comparing the clinical and/or radiographical outcomes of Ca(OH)_2_/iodoform versus ZOE pulpectomy in primary teeth of healthy children. The exclusion criteria were as follows: cross‐sectional, retrospective, laboratory, and animal studies. We also excluded all studies investigating pulpectomy in permanent teeth, traumatic teeth, or primary teeth without a succedaneous tooth. Our last exclusion criterion was any research whose study population included special needs patients.

### 2.2 Search strategy and data extraction

Search strategies were designed to identify all studies discussing the clinical and radiographical outcomes of Ca(OH)_2_/iodoform compared with ZOE used in primary teeth pulpectomy. Two commercial formulations of Ca(OH)_2_/iodoform prevail on the market, and these are Metapex (Meta Biomed Co. Ltd, Seoul, South Korea) and Vitapex (Neo Dental Chemical Products Co. Ltd, Tokyo, Japan; Nurko et al., 2000; Stuart, Schwartz, Beeson, & Owatz, 2006). Hence, the following set of keywords were used during the search: (calcium hydroxide OR Vitapex OR Metapex) AND (pulpectomy OR pulpectomies OR pulpectomized OR root canal treatment OR root canal filling) AND (primary teeth OR primary dentition OR deciduous teeth). We initially limited our search to articles published between 2003 and 2017 without restrictions on publication year or language. This search strategy yielded a total of 5,000 articles from three search engines, PubMed/MEDLINE (261), Google Scholar (3,850), and Scopus (89). Our initial search was conducted in April 2016. A subsequent search that was performed in January 2018 revealed one additional study (Chen, Liu, & Zhong, 2017) for inclusion.

The titles of all studies were reviewed by two authors independently (R. S. N. and H. J. S.). Duplicate studies were excluded. After titles selection, the abstracts were reviewed. Studies were excluded when it was obvious that the paper was not discussing any clinical and the radiographical outcomes of Ca(OH)_2_/iodoform compared with ZOE in primary teeth pulpectomy. The selected studies were downloaded as full text papers and then screened in details by the same reviewers to confirm whether they fulfilled the inclusion criteria. Cohen's *κ* statistic was done with value of 0.92 and 96.29% of agreement. Disagreement was settled by the third evaluator (A. A. E.).

Using a data extraction sheet, the reviewers next independently collected data from the selected studies. Variables included publication details (author and year), study setting, research methodology (study design, number and age of children, number of teeth, type of teeth, presence of a ZOE subgroup, ZOE, ZOE/iodoform or ZOE/iodoform combined with Ca(OH)_2_, and sample size in each group), follow‐up period(s), and clinical and radiographical outcomes. *κ* statistic was done with value of 0.82 and 98.13% of agreement. Cases of disagreement were discussed between the evaluator until agreement was reached.

In this SR, we defined (treatment) success based on the accomplishment of specific clinical and radiographical criteria. The clinical criteria are as follows: no pain, no swelling, no abscess, no pain on percussion, and/or decreased in mobility. The radiographical criteria are a decrease or an absence of radiolucency when comparing postoperative imaging with X‐rays taken preoperatively. No change in radiolucency was considered as an indicator of success in three clinical success (Chen & Liu, 2005; Gupta & Das, 2011; Subramaniam & Gilhotra, 2011). Hence, this criterion was also adopted as a measure of success in four clinical studies (Al‐Ostwani, Al‐Monaqel, & Al‐Tinawi, 2016; Pramila, Muthu, Deepa, Farzan, & Rodrigues, 2016; Trairatvorakul & Chunlasikaiwan, 2008; Xiao‐Fang & Xue‐Bin, 2003).

Authors of five included studies were contacted via email to clarify missing, unclear, or additional information (Ming‐zhi, Li, Xue‐bin, Yu‐cong, & Ting, 2009; Mortazavi & Mesbahi, 2004; Pramila et al., 2016; Ramar & Mungara, 2010; Wei‐jian, 2006), although only one responded to provide clarification of the data (Pramila et al., 2016).

### 2.3 Quality appraisal

The quality of the methodology and results of the included studies were assessed using a modified version of the Consolidated Standards of Reporting Trials (CONSORT) 2010 checklist for clinical trials quality assessment (Schulz, Altman, & Moher, 2010).

The methods and results part of CONSORT consist of 15 categories with 25 items. We added two more items, that is, the number of operators performing the pulpectomies and in studies with multiple operators, inter‐operator reliability with respect to intervention methodology and outcome measures assessed. One point was assigned per item; therefore, the scale ranged from a minimum of 0 to a maximum of 27. The reviewers then independently categorized studies according to the following scores: 19–27 indicated a low risk of bias (high‐quality study), 10–18 indicated a moderate risk of bias (moderate‐quality study), and 0–9 indicated a high risk of bias (low‐quality study). When there were discrepancies in categorization, reviewers discussed manuscript scoring until an agreement was reached. Although studies were not excluded for high bias risk, the categorizations were used for sensitivity analysis in the meta‐analysis.

The quality of each study was ranked by two independent evaluators (R. S. N. and H. J. S.). Cases of disagreement were discussed between the evaluators until agreement was reached. No exclusion based on the risk of bias was done. Studies were then classified into high, moderate, and low quality for sensitivity analysis in the meta‐analysis.

### 2.4 Statistical analysis

Studies reporting the clinical and radiographical success rates of Ca(OH)_2_/iodoform paste compared with ZOE were incorporated in the meta‐analyses using Review Manager 5.3 (The Nordic Cochrane Centre; ReviewManager [RevMan], 2014). The Mantel–Haenszel method was used to calculate a weighted average of odds ratios (ORs) and generate 95% confidence intervals (95% CIs; Landis, Sharp, Kuritz, & Koch, 2005) for the success rates of pulpectomy with Ca(OH)_2_/iodoform paste compared with ZOE across all studies. To determine whether the results of separate studies could be combined meaningfully, a statistical test of homogeneity was carried out. An inconsistency coefficient (*I* ^2^) was calculated taking into account Cochrane's heterogeneity statistic and the degrees of freedom for the sample size included in our meta‐analysis. *I* ^2^ describes the level of heterogeneity within a sample that contributes to variation as opposed to chance. The value of >25%, 50%, and 75% represent low, moderate, and high heterogeneity, respectively (Higgins, Thompson, Deeks, & Altman, 2003).

ORs were pooled with fixed effect if no heterogeneity was identified in the meta‐analysis and with random effect in case of heterogeneous studies (DerSimonian & Laird, 1986). The level of significance was set at <0.05. *Z* test was used to compare the clinical and radiographical success rates of Ca(OH)_2_/iodoform to ZOE in all follow‐up periods in high‐ and moderate‐quality studies. Success rates of high‐quality studies were compared with success rates of moderate‐quality studies using a chi‐squared test. A funnel plot was used to visually represent heterogeneity within publications; Egger's test was used for quantitative analysis of heterogeneity (Egger, Davey Smith, Schneider, & Minder, 1997). These analyses were performed using the *Comprehensive Meta*‐*Analysis* program version 3.3.070.

### 2.5 Sensitivity analysis

Meta‐analysis is confounded by many factors; these factors are thought to be a possible cause of heterogeneity if present. Subgroup analyses were used to assess the stability of the results. Analysis were carried out on the basis of the clinical and radiographical success rates to evaluate the effect of type of intracanal irrigation, type of teeth, and the quality of the studies to investigate the source of heterogeneity.

### 2.6 Level of evidence

For our SR, we developed both an evidence statement and clinical recommendations using a modification to the guidelines provided by Shekelle, Woolf, Eccles, and Grimshaw (1999). Clinical recommendations were classified on the basis of the strength of evidence by which they were supported, as determined by adherence to measurable components defined in our evidence statement. It is important to note that the classification of recommendations reflects the quality of scientific evidence supporting a given recommendation rather than its clinical importance using a system modified from that of Shekelle et al. (1999).

## 3 RESULTS

### 3.1 Study selection

**Figure 1:**
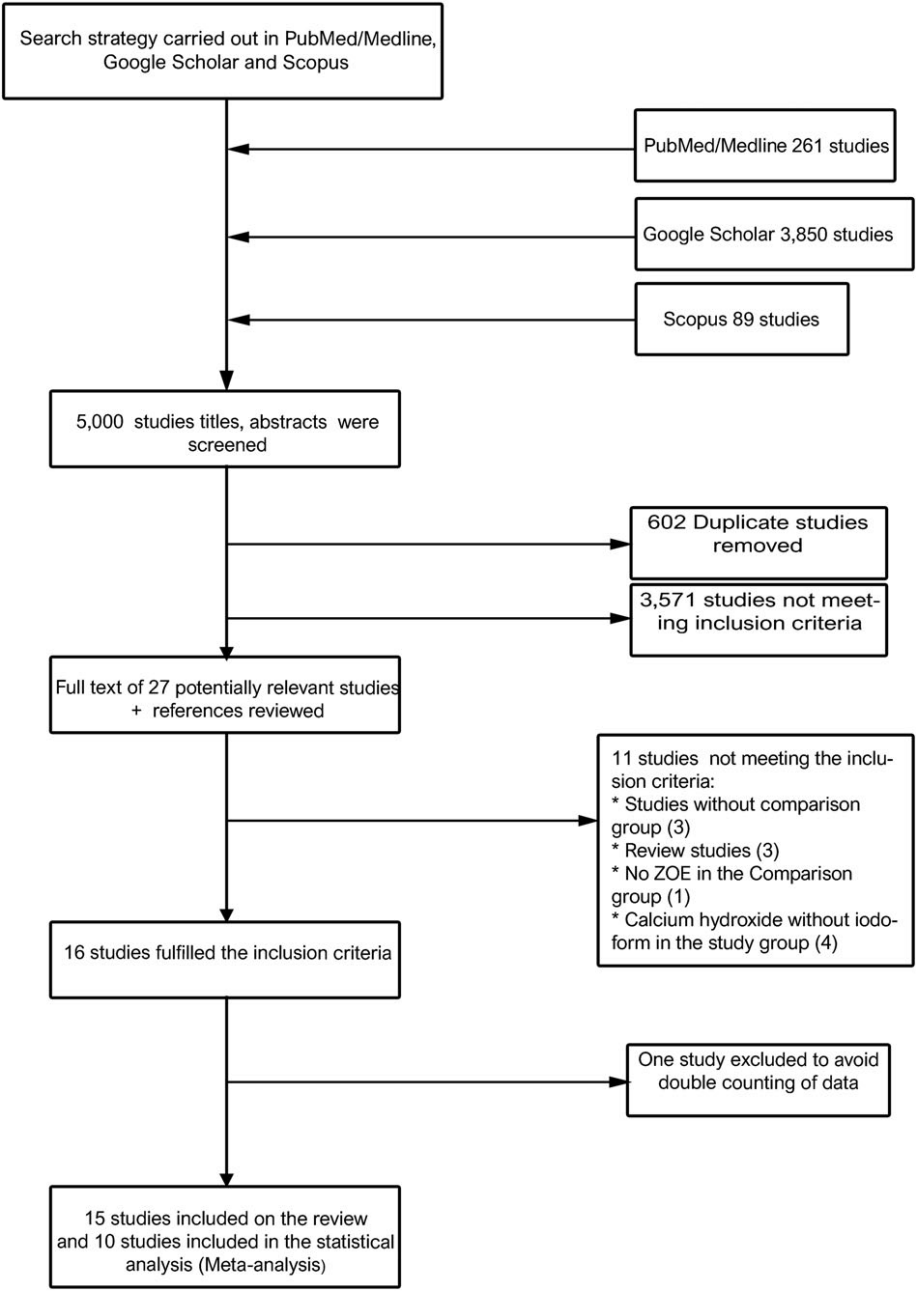
Flow diagram depicting study selection criteria. ZOE: zinc oxide eugenol

The searches yielded 5,000 potentially related titles (Figure 1). After removing the duplicate studies (602 studies) and those not eligible after reviewing the abstract, the full text of 27 studies was retrieved and compared with the inclusion criteria.

We excluded 11 studies as follows: three studies without comparison group, three were review, one study was not compared with ZOE, and four studies reported Ca(OH)_2_ without iodoform.

The total number of 16 studies were included in this SR (Figure 1). We translated seven studies into English (Chen, Lin, Zhong, & Ge, 2015; Chen & Liu, 2005; Ming‐zhi et al., 2009; Ping‐ping, 2011; Wei‐jian, 2006; Xiao‐Fang & Xue‐Bin, 2003; Yu‐xiang, Ru‐mci, & Qin, 2005) via an accredited profissional translation center. After translation, one study was excluded (Chen et al., 2015) to avoid double counting of data.

### 3.2 Characteristics of the included studies

The 15 studies included in the presented SR (Table 1) included 1,669 primary teeth (337 anterior teeth and 1,332 molars), of children aged between 3 and 13 years, pulpectomized and had follow‐up period ranged from 2 (Ozalp, Saroglu, & Sonmez, 2005; Xiao‐Fang & Xue‐Bin, 2003) to 30 months (Pramila et al., 2016) The only study with a follow‐up period shorter than 2 months reported follow‐up data after 1 week (Ping‐ping, 2011). From these studies, 11 included primary molars only (Al‐Ostwani et al., 2016; Chen et al., 2017; Gupta & Das, 2011; Ming‐zhi et al., 2009; Ozalp et al., 2005; Ping‐ping, 2011; Pramila et al., 2016; Ramar & Mungara, 2010; Subramaniam & Gilhotra, 2011; Trairatvorakul & Chunlasikaiwan, 2008; Xiao‐Fang & Xue‐Bin, 2003), and four studies included both primary incisors and molars (Chen & Liu, 2005; Mortazavi & Mesbahi, 2004; Wei‐jian, 2006; Yu‐xiang et al., 2005).

**Table 1:** Characteristics of studies included in the systematic review

Study	Site and study design	Subjects (no. of children, age in years, no. of teeth, and type of irrigation)	Follow‐up in months	Ca(OH)_2_/iodoform group			Comparison group			
				Size	Clinical success (%)	Radiograph success (%)	Type	Size	Clinical success (%)	Radiograph success (%)
Al‐Ostwani et al. (2016)^a^	University, Damascus, Syria RCT (double blinded)	39 patients 3–9 48 primary molars Sodium hypochlorite	6 12	16	15/16 (93.8) 14/16 (87.5)	12/16 (75) 12/16 (75)	ZOE	16 16	15/16 (93.8) 14/16 (87.5)	12/16 (75) 12/16 (75)
							Endoflas^b^		16/16 (100) 14/16 (87.5)	13/16 (81.3) 13/16 (81.3)
Chen and Liu (2005) a	Not mentioned, Taiwan RCT	Number of patients not mentioned 3–8 104 primary teeth: 58 primary anterior and 66 primary molars Irrigation not mentioned	18	64	45/64 (70.31) all teeth 25/30 (83.3) anterior 20/34 (58.8) molars	NM	ZOE	60	46/60 (76.66) all teeth 24/28 (85.71) anterior 22/32 (68.75) molars	NM
Chen et al. (2017)^a^	University, China RCT (double blinded)	158 patients 4–9 163 primary molars Maxillary and Mandibular: 1st and 2nd primary molars 2.5% Sodium hypochlorite	6 12 18	56	56/56 (100) 45/56 (80.4) 40/56 (71.4)	53/56 (94.5) 34/56 (60.7) 30/56(53.6)	ZOE	51	51/51 (100) 51/51 (100) 47/51(92)	51/51 (100) 51/51 (100) 45/51 (88)
							MPRCF^b^	53	53/53 (100) 53/53 (100) 51/53 (96)	53/53 (100) 53/53 (100) 49/53 (92)
Gupta and Das (2011)^a^	University, Kolkata, India CT	34 patients 4–7 42 primary mandibular molars Sodium hypochlorite + saline	3 6	21	20/21 (95) 19/21 (90)	20/21 (95) 20/21 (95)	ZOE	21	18/21 (85.7) 18/21 (85.7)	19/21 (90) 19/21(90)
Ming‐zhi et al. (2009)	University, China RCT	115 patients 5–9 150 primary molars Hydrogen peroxide	6	66	Total success 55/66 (83.33)	NM	ZOE	58	NM	NM
Mortazavi and Mesbahi (2004)	Not mentioned, Iran RCT	58 Patients 3–13 58 primary teeth: 53 maxillary and mandibular primary molars and 5 primary anterior Saline	3 10–16	26	26/26 (100) 13/13 (100)	NM 26/26 (100)	ZOE	32	32/32 (100) 28/32 (87.5)	NM 28/32 (87.5)
Ozalp et al. (2005)^a^	University, Turkey RCT (single blinded)	76 patients 4–9 40 primary molars Maxillary and mandibular primary molars: 1st and 2nd molars Sodium hypochlorite + metronidazole	2 4 6 8 10 12 18	20	20/20 (100) 20/20 (100) 20/20 (100) 20/20 (100) 20/20 (100) 20/20 (100) 20/20 (100)	20/20 (100) 20/20 (100) 20/20 (100) 20/20 (100) 20/20 (100) 20/20 (100) 20/20 (100)	ZOE	20	20/20 (100) 20/20 (100) 20/20 (100) 20/20 (100) 20/20 (100) 20/20 (100) 20/20 (100)	20/20 (100) 20/20 (100) 20/20 (100) 20/20 (100) 20/20 (100) 20/20 (100) 20/20 (100)
Ping‐ping (2011)	Hospital, China RCT	50 patients 10–13 60 primary molars Saline + 3% hydrogen peroxide	1 week	30	29/30 (96.66)	NM	ZOE/iodoform	30	17/30 (56.66)	NM
Pramila et al. (2016)^a^	College and hospital, India RCT (double blinded)	88 patents 4–9 129 primary mandibular molars 1st and 2nd molars Saline + chlorhexidine	6 12 30	43	35/35 (100) 28/28 (100) 29/29 (100)	30/35 (85.8) 25/28 (89.2) 28/29 (96.5)	ZOE/iodoform	43	35/35 (100) 32/32 (100) 31/31 (100)	32/35 (91.4) 31/32 (94) 30/31 (96.77)
							ZOE	43	36/36 (100) 32/32 (100) 30/30 (100)	36/36 (100) 32/32 (100) 30/30 (100)
Ramar and Mungara (2010)	College and hospital, India CT	77 patients 4–7 96 primary mandibular molars Sodium hypochlorite + chlorohexidine	3 6 9	30	30/30 (100) 30/30 (100) 30/30 (100)	30/30 (100) 30/30 (100) 26/26 (100)	ZOE/iodoform	34	34/34 (100) 33/34 (97) 31/31 (100)	32/34 (94.11) 27/34 (79.11) 31/31 (100)
							Endoflas^b^	32	32/32 (100) 32/32 (100) 31/31 (100)	32/32 (100) 32/32 (100) 32/32 (100)
Subramaniam and Gilhotra (2011)^a^	College, hospital, and research center, Bangalore RCT	Number of patients not mentioned 5–9 45 primary teeth: 5 maxillary, 40 mandibular Primary molars: 1st and 2nd molars Saline + sodium hypochlorite	3 6 12 18	15	15/15 (100) 15/15 (100) 15/15 (100) 15/15 (100)	15/15 (100) 15/15 (100) 15/15 (100) 15/15 (100)	ZOE	15	14/15 (93.3) 14/15 (93.3) 14/15 (93.3) 14/15 (93.3)	14/15 (93.3) 14/15 (93.3) 14/15 (93.3) 14/15 (93.3)
							Endoflas^b^	15	14/15 (93.3) 14/15 (93.3) 14/15 (93.3) 14/15 (93.3)	14/15 (93.3) 14/15 (93.3) 14/15 (93.3) 14/15 (93.3)
Trairatvorakul and Chunlasikawan (2008)^a^	Not mentioned ,Thailand RCT	42 patients 3.4–7.9 54 primary mandibular molars 1st and 2nd molars Sodium hypochlorite	6 12	27	27/27 (100) 26/27 (96)	21/27 (78) 24/27 (89)	ZOE	27	26/27 (96) 25/27 (93)	23/27 (85) 24/27 (88.8)
Wei Jian (2006)^a^	Hospital, China CT	179 patients 3–10 283 primary teeth: 79 anterior, 23 canine, and 181 molars Hydrogen peroxide + saline	3 12	87	86/87 (98.8) 84/87 (96.5)	NM	ZOE	196	194/196 (98.9) 190/196 (96.9)	NM
Xiao‐Fang and Xue‐ Bin (2003)^a^	Hospital, China CT	72 patients 4–9 81 primary molars: 37 maxillary and 44 mandibular Primary molars: 1st and 2nd molars Irrigation material not mentioned	2 4 6	39	There were no clear data about the overall clinical success	20/20 (100) 2/20 (100) 19/20 (95)	ZOE	42	There were no clear data about the overall clinical success	15/17 (88) 15/17 (88) 16/17 (94)
Yu‐xiang et al. (2005)	Hospital, China RCT	273 patients 296 teeth 162 incisors 10 canines 124 Molars 1st and 2nd molars Irrigation material not mentioned	12	151	NM	NM	ZOE/iodoform	145	NM	NM

*Note*. CT: clinical trials; NM: not mentioned; RCT: randomized controlled trials; ZOE: zinc oxide eugenol.

a Studies included in meta‐analysis.

b Endoflas and MPRCF: ZOE/iodoform and Ca(OH)_2_.

Only the aforementioned Ca(OH)_2_/iodoform products, Metapex and Vitapex, were used in these studies; Metapex was used in four studies (Al‐Ostwani et al., 2016; Gupta & Das, 2011; Ramar & Mungara, 2010; Subramaniam & Gilhotra, 2011), whereas Vitapex was used in 10 studies (Chen et al., 2017; Chen & Liu, 2005; Ming‐zhi et al., 2009; Mortazavi & Mesbahi, 2004; Ozalp et al., 2005; Pramila et al., 2016; Trairatvorakul & Chunlasikaiwan, 2008; Wei‐jian, 2006; Xiao‐Fang & Xue‐Bin, 2003; Yu‐xiang et al., 2005), and one study mentioned using Ca(OH)_2_/iodoform without mentioning its manufacturer (Ping‐ping, 2011).

The included studies had different eligibility criteria as well as different study methodologies. Variations were present in the number of treatment visits, the latency to follow‐up examination, the type of irrigation solution used, and the final restorative material used (Table 1). Ca(OH)_2_/iodoform paste was compared with ZOE in 8 studies (Chen & Liu, 2005; Gupta & Das, 2011; Ming‐zhi et al., 2009; Mortazavi & Mesbahi, 2004; Ozalp et al., 2005; Trairatvorakul & Chunlasikaiwan, 2008; Wei‐jian, 2006; Xiao‐Fang & Xue‐Bin, 2003), ZOE/ iodoform in two studies (Ping‐ping, 2011; Yu‐xiang et al., 2005), ZOE and ZOE/iodoform combined with Ca(OH)_2_ in three studies (Al‐Ostwani et al., 2016; Chen et al., 2017; Subramaniam & Gilhotra, 2011), ZOE and ZOE/iodoform in one study (Pramila et al., 2016), and ZOE/iodoform and ZOE/iodoform combined with Ca(OH)_2_ in one study (Ramar & Mungara, 2010).

Across all studies, the clinical success rates were as follows: 70–100% for Ca(OH)_2_/iodoform, 77–100% for ZOE, 57–100% for ZOE/iodoform, and 88–100% for ZOE/iodoform/Ca(OH)_2_. The radiographical success rates were 61–100% for Ca(OH)_2_/iodoform,75–100% for ZOE, 79–100% for ZOE/iodoform, and 81–100% for ZOE/iodoform with Ca(OH)_2_.

### 3.3 Quality assessment

Eleven of the included studies were randomized clinical trials (Al‐Ostwani et al., 2016; Chen et al., 2017; Chen & Liu, 2005; Ming‐zhi et al., 2009; Mortazavi & Mesbahi, 2004; Ozalp et al., 2005; Ping‐ping, 2011; Pramila et al., 2016; Subramaniam & Gilhotra, 2011; Trairatvorakul & Chunlasikaiwan, 2008; Yu‐xiang et al., 2005), three of them were double‐blinded (Al‐Ostwani et al., 2016; Chen et al., 2017; Pramila et al., 2016), and one was single‐blinded (Ozalp et al., 2005; Table 2). Two studies reported the methodology by which sample size was determined (Chen et al., 2017; Pramila et al., 2016). Using our modified CONSORT 2010 checklist, only two studies were determined to have low risk of bias (Chen et al., 2017; Pramila et al., 2016; high quality). Eleven studies were shown to have a moderate risk of bias (Al‐Ostwani et al., 2016; Chen & Liu, 2005; Gupta & Das, 2011; Mortazavi & Mesbahi, 2004; Ozalp et al., 2005; Ramar & Mungara, 2010; Subramaniam & Gilhotra, 2011; Trairatvorakul & Chunlasikaiwan, 2008; Wei‐jian, 2006; Xiao‐Fang & Xue‐Bin, 2003; Yu‐xiang et al., 2005; moderate quality), and two studies were considered to have a high risk of bias (Ming‐zhi et al., 2009; Ping‐ping, 2011; low quality). The high‐quality studies received similar quality scores, differing by only one point; this occurred because the lower scoring study (Pramila et al., 2016) provided a less detailed explanation of the outcome measure. The moderate‐quality studies most often received lower scores due to an omission of sample size, an unclear design of the study, and lack of randomization implementation. Similarly, the low‐quality studies scored poorly in multiple categories for a variety of reasons, including unclear study design, lack of randomization implementation, failing to randomize subjects upon study administration despite having proposed randomization, lack of blinding, and lack of inter‐operator reliability (Table 2).

**Table 2:** Studies' quality assessment

Topic (points) /study	Chen et al. (2017)*	Pramila et al. (2016)*	Trairatvorakul and Chunlasikaiwan (2008)*	Al‐Ostwani et al. (2016)*	Ozalp et al. (2005)*	Yu‐xiang et al. (2005)	Xiao‐Fang and Xue‐Bin (2003)*	Mortazavi and Mesbahi (2004)	Subramaniam and Gilhotra (2011)*	Chen and Liu (2005)*	Ramar and Mungara (2010)	Wei‐jian (2006)*	Gupta and Das (2011)*	Ming‐zhi et al. (2009)	Ping‐ping (2011)
Method															
Trial design (2)	2	2	2	0	0	1	0.5	2	0	1	0	1	0	0	0
Participants (2)	2	2	1	1.5	1.5	2	2	1	1.5	1	1.5	2	1.5	1	1
Interventions (2)	2	2	2	2	2	2	1	1	2	2	2	2	0.5	1	1
Outcomes (1)	1	1	1	1	1	1	1	1	1	0.5	1	0.5	1	1	0.5
Sample size (2)	2	2	0	0	0	0	0	0	0	0	0	0	0	0	0
Randomization (1)	1	1	1	1	1	1	1	0	1	1	0	1	0	1	1
Sequence generation (2)	2	2	1	0	0	0	0	2	0	0	1	0	0	0	0
Allocation and concealment (1)	1	1	1	0	0	0	0	1	0	0	0	0	0	0	0
Implementation (1)	1	1	1	0	0	0	0	0	0	0	0	0	0	0	0
Blinding (2)	2	2	0	2	1.5	0	0	0	0	0	0	0	0	0	0
Results															
Statistical methods (2)	2	1.5	1	1	1	1	1	1	1	2	1	0	0	1	0
Participant (2)	2	2	1.5	1.5	1.5	0.5	1	1	1	1.5	0.5	0.5	2	1	0.5
Recruitment (2)	2	2	1.5	2	2	2	1	0.5	2	1	2	2	2	1	1
Baseline data (1)	1	1	0.5	0	1	1	1	1	0.5	0	0.5	0	1	0.5	1
Numbers analyzed (1)	1	1	1	1	0.5	1	0.5	0	1	0.5	0.5	1	1	0.5	1
Outcomes (1)	2	0.5	0.5	0.5	0.5	0	0.5	0	0.5	0	0	0	0.5	0.5	0.5
Reliability + number of operator (2)	2	2	1	1	1	0	2	0.5	0	0.5	1	0	0	0	0
Total/27 Quality	27 High	26 High	17 Moderate	14.5 Moderate	14.5 Moderate	12.5 Moderate	12.5 Moderate	12 Moderate	11.5 Moderate	11 Moderate	11 Moderate	10 Moderate	9.5 Moderate	8.5 Low	7.5 Low

* Studies included in the meta‐analysis.

### 3.4 Meta‐analysis

Of the 15 included studies in the systematic rivew, 10 were included in the meta‐analysis (Al‐Ostwani et al., 2016; Chen et al., 2017; Chen & Liu, 2005; Gupta & Das, 2011; Ozalp et al., 2005; Pramila et al., 2016; Subramaniam & Gilhotra, 2011; Trairatvorakul & Chunlasikaiwan, 2008; Wei‐jian, 2006; Xiao‐Fang & Xue‐Bin, 2003). Five studies were excluded from the analysis because of missing or wrong data. In the meta‐analysis, the comparison group was subgrouped into ZOE, ZOE/iodoform, and ZOE/iodoform combined with Ca(OH)_2_.

**Figure 2:**
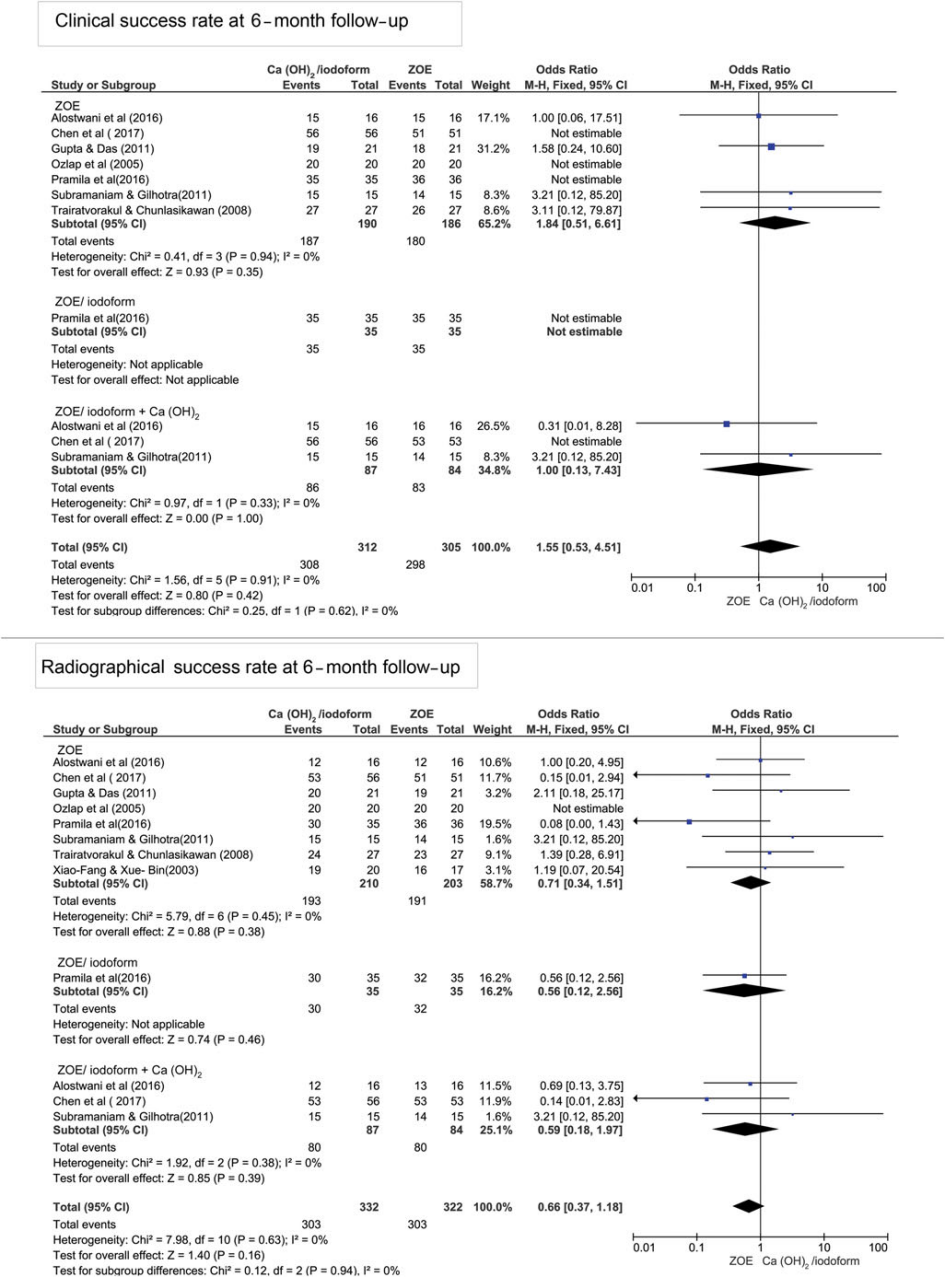
Forest plot for meta‐analysis of the clinical and radiographical success rates of Ca(OH)_2_/iodoform pulpectomy compared with zinc oxide eugenol (ZOE), ZOE/iodoform, and ZOE/iodoform combined with Ca(OH)_2_ at 6‐month follow‐up. CI: confidence interval

At 6‐month follow‐up, there was no statistically significant difference in the clinical and radiographical success rates between Ca(OH)_2_/iodoform compared with ZOE (clinical *P* = 0.35, OR: 1.84, and 95% CI: 0.51–6.61 and radiographical *P* = 0.38, OR: 0.71, and 95% CI: 0.34–1.51), ZOE/iodoform (clinical did not show any estimable results and radiographical *P* = 0.46, OR: 0.56, and 95% CI: 0.12–2.56), and ZOE/iodoform combined with Ca(OH)_2_ (clinical *P* = 1.00, OR: 1.00, and 95% CI: 0.13–7.43 and radiographical *P* = 0.39, OR: 0.59, and 95% CI: 0.18 to 1.97; Figure 2).

**Figure 3:**
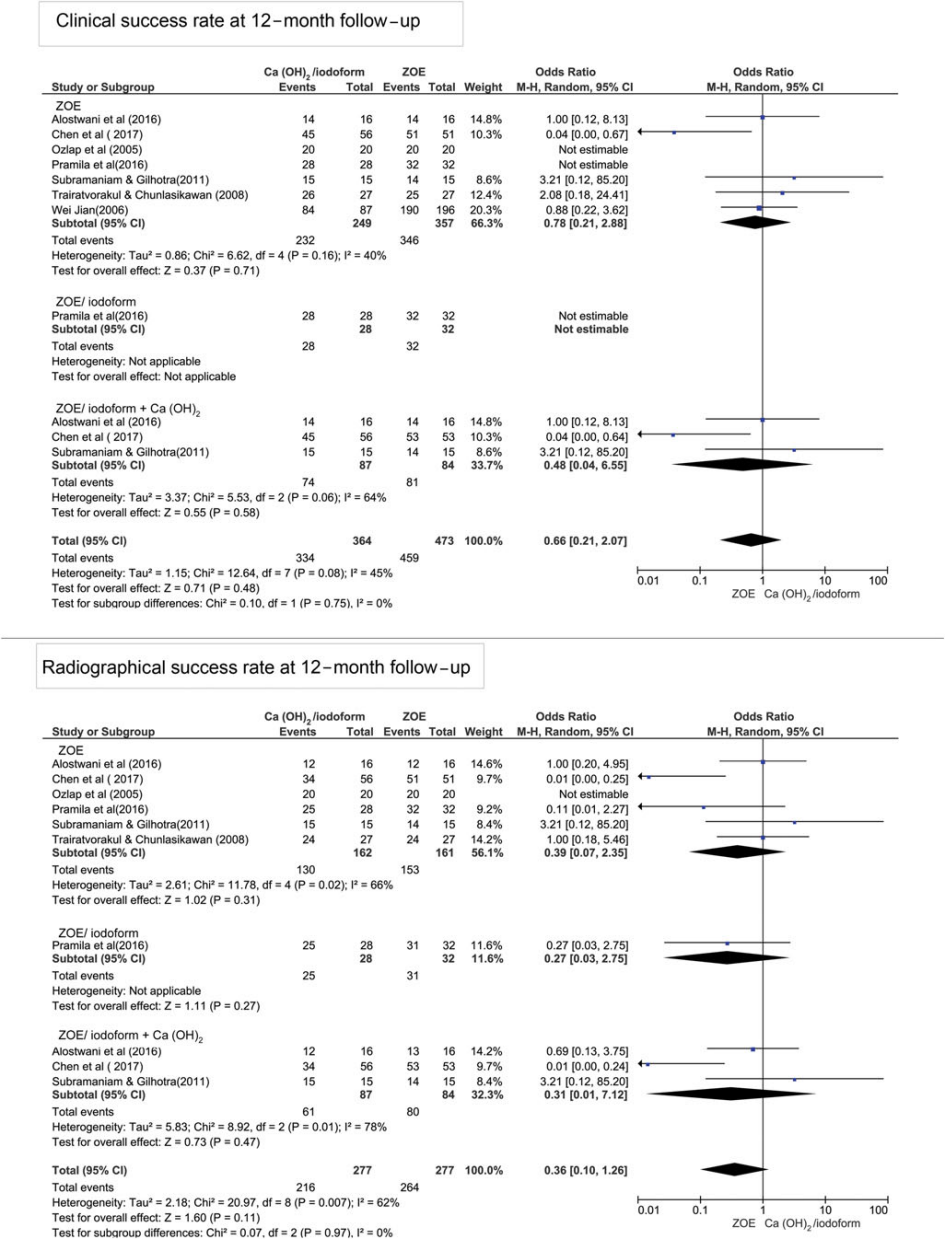
Forest plot for meta‐analysis of the clinical and radiographical success rates of Ca(OH)_2_/iodoform pulpectomy compared with zinc oxide eugenol (ZOE), ZOE/iodoform, and ZOE/iodoform combined with Ca(OH)_2_ at 12‐month follow‐up. CI: confidence interval

At 12‐month follow‐up, Figure 3 shows that there was no statistically significant difference in terms of clinical and radiographical success rates between Ca(OH)_2_/iodoform compared with ZOE (clinical *P* = 0.07, OR: 0.78, and 95% CI: 0.21–2.88 and radiographical *P* = 0.31, OR: 0.39, and 95% CI: 0.07–2.35), ZOE/iodoform (clinical did not show any estimable results and radiographical *P* = 0.27, OR: 0.27, and 95% CI: 0.03–2.75), and ZOE/iodoform combined with Ca(OH)_2_ (clinical *P* = 0.58, OR: 0.48, and 95% CI: 0.04–6.55 and radiographical *P* = 0.47, OR: 0.31, and 95% CI: 0.01 to 7.12).

**Figure 4:**
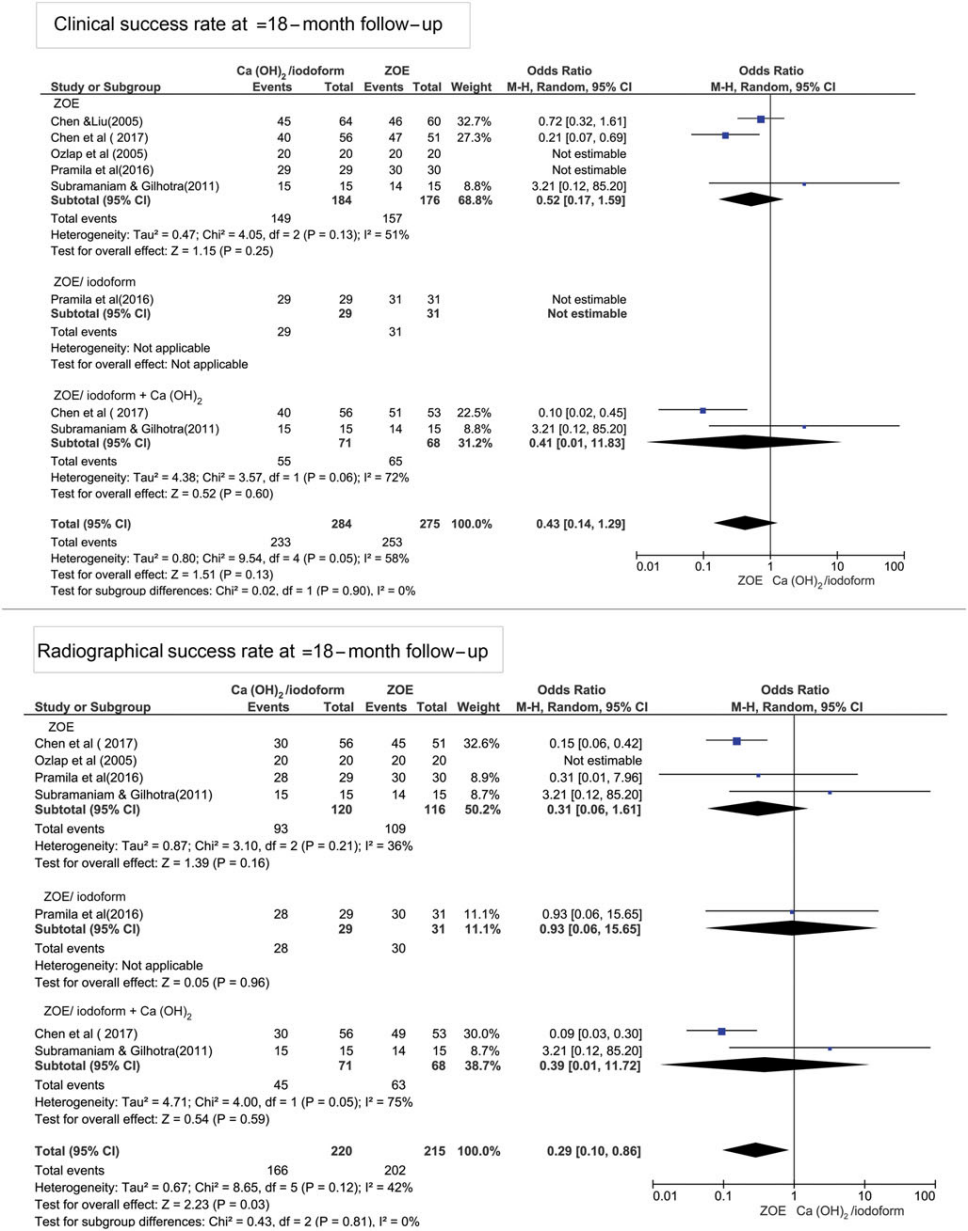
Forest plot for meta‐analysis of the clinical and radiographical success rates of Ca(OH)_2_/iodoform pulpectomy compared with zinc oxide eugenol (ZOE), ZOE/iodoform, and ZOE/iodoform combined with Ca(OH)_2_ at ≥18‐month follow‐up

At ≥18‐month follow‐up period, Figure 4 shows that there was no statistically significant difference in the clinical and radiographical success rates between Ca(OH)_2_/iodoform compared with ZOE (clinical *P* = 0.25, OR: 0.52, and 95% CI: 0.17–1.59 and radiographical *P* = 0.16, OR: 0.13, and 95% CI: 0.06–1.61), ZOE/iodoform (clinical did not show any estimable results and radiographical *P* = 0.96, OR: 0.93, and 95% CI: 0.06–15.65), and ZOE/iodoform combined with Ca(OH)_2_ (clinical *P* = 0.60, OR: 0.41, and 95% CI: 0.14–1.29 and radiographical *P* = 0.59, OR: 0.39, and 95% CI: 0.01–11.72).

Our meta‐analysis also investigated the effect of confounding factors on the clinical and radiographical success rates of Ca(OH)_2_ compared with ZOE and ZOE/iodoform combined with Ca(OH)_2_. In the subgroup analysis, we excluded one study that reported the success rates of ZOE/iodoform because there was no sufficient data for comparison (Pramila et al., 2016). Possible confounders included intracanal irrigation, type of molars, and study quality.

Because studies reported the use of different intracanal irrigation materials, they were further subdivided into two groups: those in which sodium hypochlorite (NaOCl) was used and studies in which any other intracanal irrigation was used. We compared the effect of varying intracanal irrigation solutions only to the ZOE group, because the ZOE/iodoform with Ca(OH)_2_ did not have enough data for the comparison. There was no statistically significant difference between the two groups of studies at 6‐, 12‐, and ≥18‐month period when using Ca(OH)_2_/iodoform compared with ZOE group (*P* > 0.05) for both clinical and radiographical success rates (Figures S1–S3).

Studies were subgrouped according to the types of molars included in their studies: mandibular molars compared with maxillary and mandibular molars. We compared the effect of the type of molars in ZOE group only, because the ZOE/iodoform combined with Ca(OH)_2_ had no enough data for the comparison. There were no statistically significant differences between the subgroups of studies at all follow‐up periods (*P* > 0.05; Figures S4–S6).

The 10 studies included in the meta‐analysis were either moderate‐ (eight studies) or high‐quality (two studies; Table 2). At 6‐month follow‐up, the two high‐quality studies (Chen et al., 2017; Pramila et al., 2016) had 100% clinical success rates in all groups of the study (Ca(OH)_2_/iodoform paste, ZOE, and ZOE/iodoform combined with Ca(OH)_2_). On the other hand, the clinical success rates of the moderate‐quality studies averaged 93.8–100%, 85–100%, and 93–100% for the Ca(OH)_2_/iodoform, ZOE, and ZOE/iodoform/Ca(OH)_2_ subgroups, respectively. The moderate‐quality studies showed no statistically significant difference between either ZOE (*P* = 0.35, OR: 1.84, and 95% CI: 0.51–6.61) or ZOE/iodoform with Ca(OH)_2_ (*P* = 1.00, OR: 1.00, and 95% CI: 0.13–7.43) compared with Ca(OH)_2_/iodoform (Figure S7).

The radiographical success rates between Ca(OH)_2_/iodoform paste and ZOE and ZOE/iodoform combined with Ca(OH)_2_ at 6‐month period in relation to studies quality was evaluated. The ZOE showed statistically significant higher success rates in high‐quality studies compared with Ca(OH)_2_/iodoform (*P* = 0.03, OR: 0.10, and 95% CI: 0.01–0.83). However, no statistically significant difference was noticed on high‐quality studies when comparing Ca(OH)_2_/iodoform to ZOE/iodoform combined with Ca(OH)_2_ (*P* = 0.20, OR: 0.14 and 95% CI: 0.01–2.83). The high‐quality studies revealed a higher statistically significant difference than the moderate‐quality studies when comparing Ca(OH)_2_/iodoform to ZOE (*P* = 0.03) and no significant difference when comparing the Ca(OH)_2_/iodoform to ZOE/iodoform combined with Ca(OH)_2_ (*P* = 0.20; Figure S8).

**Figure 5:**
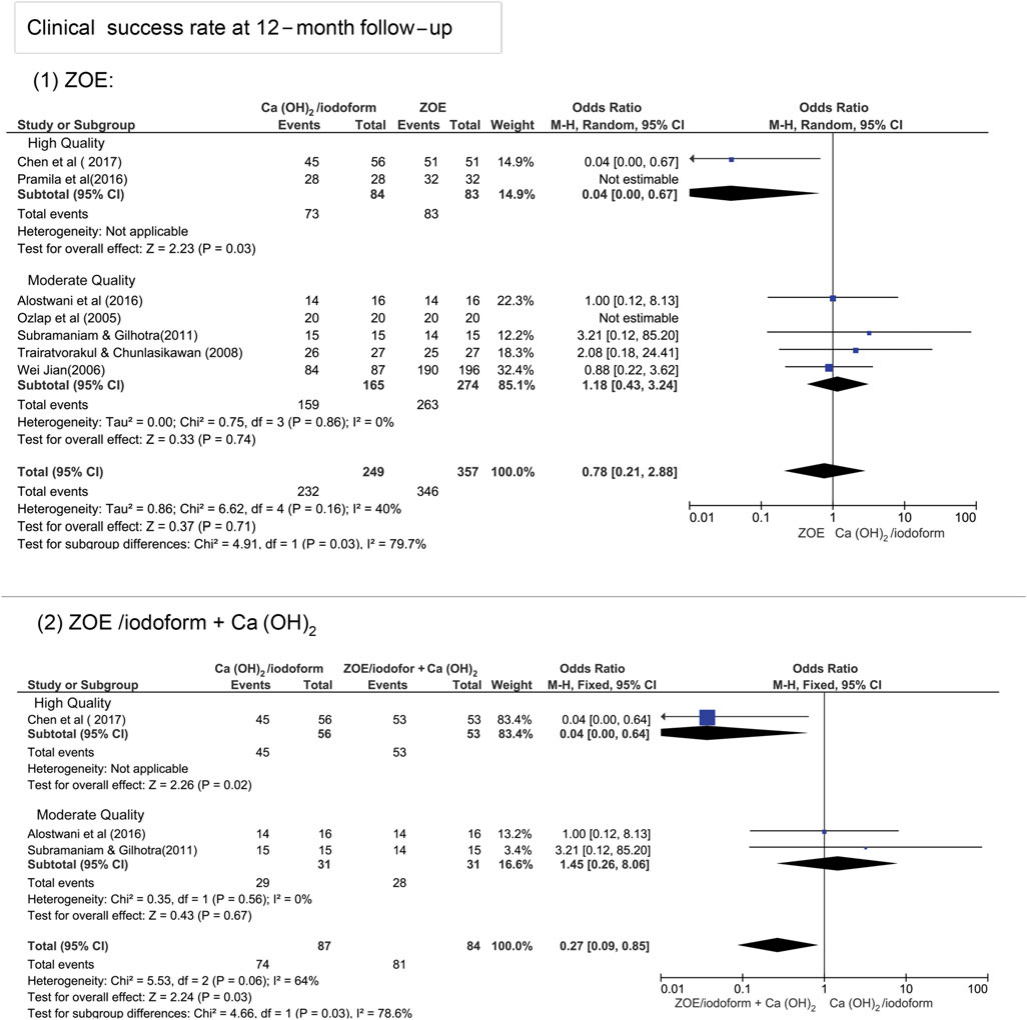
Forest plot for meta‐analysis of the clinical success rates of Ca(OH)_2_/iodoform compared with zinc oxide eugenol (ZOE) and ZOE/iodoform combined with Ca(OH)_2_ at 12‐month follow‐up within studies of high and moderate quality

**Figure 6:**
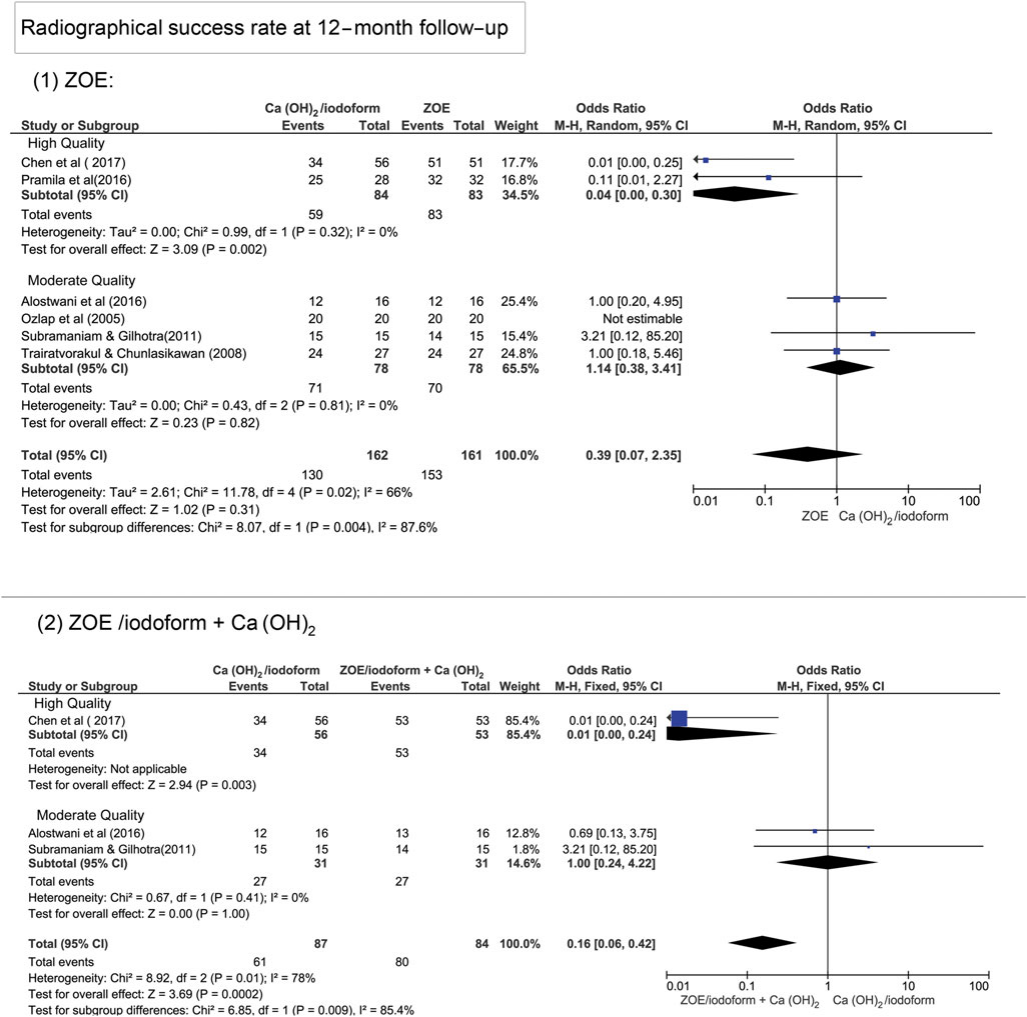
Forest plot for meta‐analysis of the radiographical success rates of Ca(OH)_2_/iodoform compared with zinc oxide eugenol (ZOE) and ZOE/iodoform combined with Ca(OH)_2_ at 12‐month follow‐up within studies of high and moderate quality

At the 12‐month period in relation to studies' quality, the high‐quality studies show statistically significant higher clinical and radiographical success rates in ZOE and ZOE/iodoform combined with Ca(OH)_2_ compared with Ca(OH)_2_/iodoform (*P* < 0.05). Although the moderate‐quality studies show no statistically significant difference between Ca(OH)_2_/iodoform compared with either ZOE or ZOE/iodoform combined with Ca(OH)_2_ in both clinical and radiographical success rates (*P* > 0.05). There was statistically significant difference in clinical and radiographical success rates between high‐ and moderate‐quality studies when comparing Ca(OH)_2_/iodoform with ZOE and ZOE/iodoform combined with Ca(OH)_2_ (*P* < 0.05; Figures 5 and 6).

**Figure 7:**
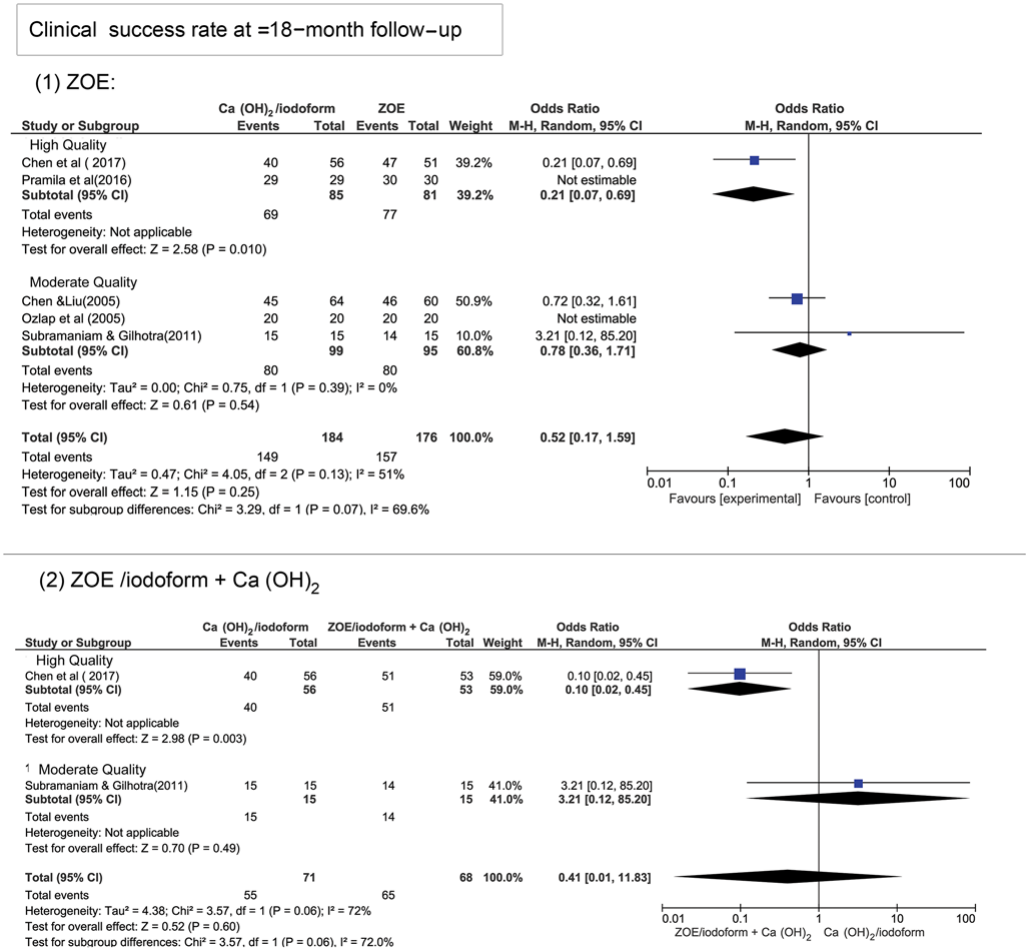
Forest plot for meta‐analysis of the clinical success rates of Ca(OH)_2_/iodoform compared with zinc oxide eugenol (ZOE) and ZOE/iodoform combined with Ca(OH)_2_ at ≥18‐month follow‐up within studies of high and moderate quality

**Figure 8:**
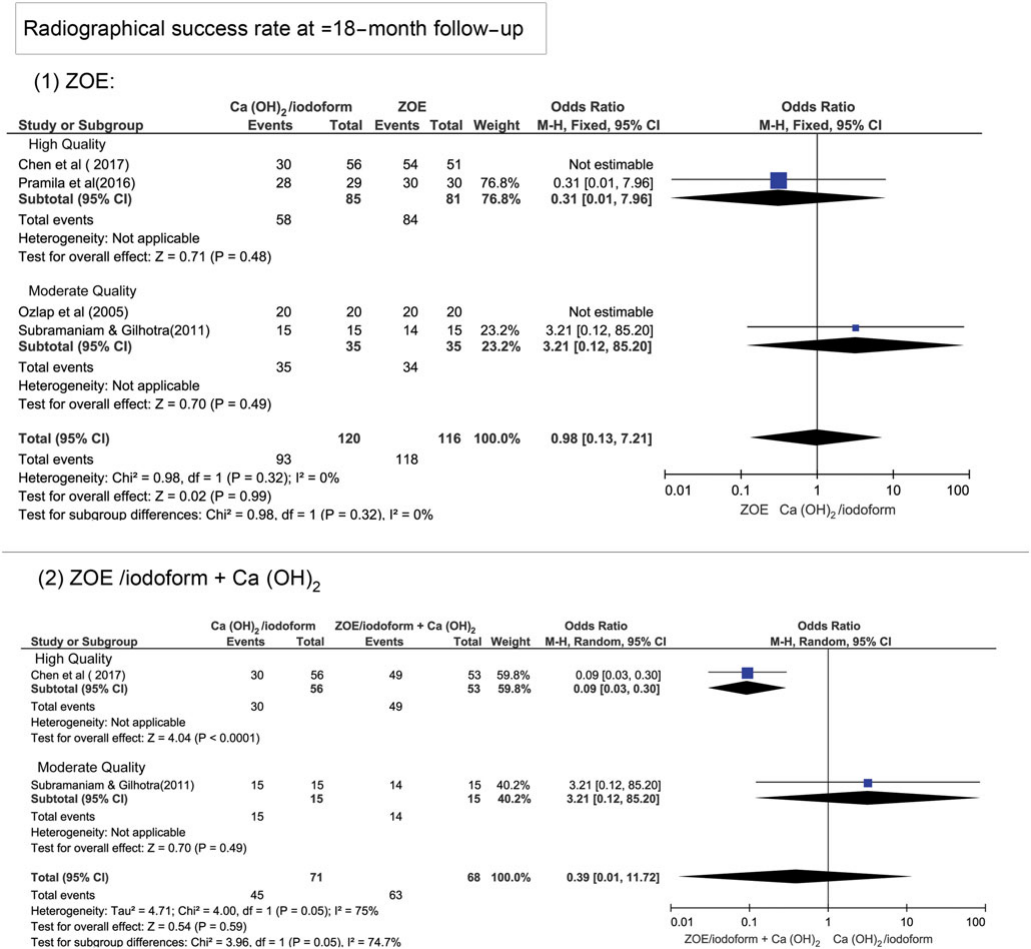
Forest plot for meta‐analysis of the radiographical success rates of Ca(OH)_2_/iodoform compared with ZOE and ZOE/iodoform combined with Ca(OH)_2_ at ≥18‐month follow‐up within studies of high and moderate quality

At ≥18‐month period, the high‐quality studies demonstrated higher clinical success rates when comparing Ca(OH)_2_/iodoform to ZOE (*P* = 0.010, OR: 0.21, and 95% CI: 0.07–0.69) and ZOE/iodoform combined to Ca(OH)_2_ (*P* = 0.003, OR: 0.10, and 95% CI: 0.02–0.45). No statistically significant difference was noticed between Ca(OH)_2_/iodoform compared with ZOE and ZOE/iodofom combined with Ca(OH)_2_ in moderate‐quality studies (*P* > 0.05). Also, no statistically significant difference present regarding the clinical success rates when comparing high‐ to moderate‐quality studies (*P* > 0.05; Figure 7). In terms of radiographical success rates, the high‐quality studies demonstrated higher success rates when comparing Ca(OH)_2_/iodoform with ZOE/iodoform combined with Ca(OH)_2_ (*P* = 0.0001, OR: 0.09 and 95% CI: 0.03–0.30), whereas no statistically significant difference was noticed when comparing Ca(OH)_2_/iodoform with ZOE (*P* = 0.48, OR: 0.31, and 95% CI: 0.01–7.96). In moderate‐quality studies, no statistically significant difference in the radiographical success rates between Ca(OH)2/iodoform compared with ZOE and ZOE/iodoform combined with Ca(OH)_2_ (*P* > 0.05; Figure 8).

### 3.5 Heterogeneity

Strong evidence of heterogeneity was observed in the clinical success rates at 12 (*I* ^2^ = 45%) and ≥18 (*I* ^2^ = 58%) months and radiographical success rates at 12 (*I* ^2^ = 62%) and ≥18 (*I* ^2^ = 42%) months of follow‐up. To explore this heterogeneity, a funnel plot was generated. At 12 and ≥18 months, both clinical and radiographical success rates on the graphs showed an asymmetry indicating that this heterogeneity may be due to chance.

### 3.6 Evaluation of small study effects

Funnel plots were used for all studies together evaluating the success rates between Ca(OH)_2_/iodoform compared with ZOE, ZOE/iodoform, and ZOE/iodoform combined with Ca(OH)_2_. Absence of small study effect was found as the graphs had the shape of a funnel, and the studies were almost symmetrical around the central line at 6 months both for clinical and radiographical success rates. Conversely, funnel plots evaluating the 12‐ and ≥18‐month clinical and radiographical success rates on the graphs showed an assymetry, indicating the presence of publication bias (Sedgwick, 2013; Figure S9).

Egger's test was conducted to quantitatively determine asymmetry around central lines in generated funnel plots, thereby allowing us to further investigate whether small study effects were present. No statistically significant small study effect was detected at 6 months regarding clinical and radiographical success rates (clinical *P* = 0.93 and radiographical *P* = 0.58), 12 months clinical and radiographical success rates (clinical *P* = 0.66 and radiographical *P* = 0.30), and clinical success rates at ≥18 months (*P* = 0.79). However, a quantitative asymmetry was observed in the funnel plot depicting radiographical success rates at ≥18‐month follow‐up, indicating statistically significant small study effects (*P* = 0.02).

### 3.7 Level of evidence

Because there were no overall differences in clinical or radiographical success rates ≥18‐month post‐procedurally, Ca(OH)_2_/iodoform, ZOE, and ZOE/iodoform combined with Ca(OH)_2_ may be used interchangeably for the pulpectomy of primary teeth (level of evidence Ib, grade A recommendation). However, in young children with teeth expected to have a longer life span, it is recommended to use ZOE or ZOE/iodoform combined with Ca(OH)_2_ (evidence level Ia) and strength of recommendation level (A). However, conclusions drawn from the two high‐quality studies analyzed indicate that in young children, the use ZOE or ZOE/iodoform combined with Ca(OH)_2_ is recommended (level of evidence Ia, grade A recommendation).

## 4 DISCUSSION

Our meta‐analysis was the first ever to compare Ca(OH)_2_/iodoform and ZOE used in primary teeth pulpectomy. This SR included 15 recent studies with no limitation in time and language. Out of them, 10 studies were included in the meta‐analysis. This SR found no statistically significant difference on the clinical and radiographical success rate of Ca(OH)_2_/iodoform compared with ZOE, ZOE/iodoform, and ZOE/iodoform combined with Ca(OH)_2_ used in primary teeth pulpectomy up to ≥18‐month follow‐up period.

Intracanal irrigations have the potential to alter the success rates of primary teeth pulpectomies. Pozos‐Guillen, Garcia‐Flores, Esparza‐Villalpando, and Garrocho‐Rangel (2016) conducted an SR and meta‐analysis and reported the clinical, radiographical, and microbiological results of intracanal irrigations for primary teeth pulpectomy. Similar to the results of our study, they found that different intracanal irrigation materials did not differ in their ability to reduce bacterial count in the root canal. They reported that the evidence was inconclusive as to which intracanal irrigant would be ideally utilized (Pozos‐Guillen et al., 2016). We determined that there was no statistically significant difference between NaOCl and other irrigation solutions used in primary teeth pulpectomy regardless of the filling material utilized.

A second factor potentially affecting success rates of primary teeth pulpectomies was the type of tooth operated on. To date, there have been no studies comparing the clinical and radiographical success rates of primary teeth pulpectomy in relation to the type of teeth on which procedures were performed. However, some researchers prefer including only mandibular molars to facilitate the identification of furcation pathosis and determine the rate of healing (Pramila et al., 2016; Trairatvorakul & Chunlasikaiwan, 2008). Barja‐Fidalgo, Moutinho‐Ribeiro, Oliveira, and de Oliveira (2011) investigated permanent teeth pulpectomy success rates and revealed that there was no difference in outcomes for maxillary or mandibular teeth. Expanding upon these results, our study determined that pulpectomy success rates in primary teeth were also not affected by tooth type.

When stratifying our meta‐analysis by study quality, differing results were uncovered. We found that ZOE use was associated with a statistically significantly higher success rates than Ca(OH)_2_/iodoform in high‐quality studies. This difference, however, was not present in moderate‐quality studies. This difference could be a consequence of the limitations found in moderate‐quality studies such as small sample size, lack of sample size calculation, the unclear design of the study, and limited time of follow‐up.

According to Al‐Namankany, Ashley, Moles, and Parekh (2009) and Rajasekharan, Vandenbulcke, and Martens (2015), the quality of reporting randomized clinical trials in pediatric dentistry journals was poor and inadequate for ensuring reliable and reproducible results. In addition, the CONSORT group reported that meta‐analyses including low‐quality randomized clinical trials may overestimate success rates of a given medical intervention by 35% in Medicine (Moher et al., 1998; Schulz, Chalmers, Hayes, & Altman, 1995). We believe that our subgroup analysis comparing success rates within studies of high and moderate quality provides additional information that remains uninfluenced by research with a high risk of bias.

This SR and meta‐analysis had some limitations. For example, we observed moderate to high levels of heterogeneity across included studies. Specifically, a moderate level of heterogeneity was found in the 12‐ and ≥18‐month follow‐up. This may have stemmed from systematic differences within the studies analyzed; that is, different eligibility criteria yielding distinct patient populations, varying levels of and rationale for participant dropout, varying methods used to evaluate radiographical success rates, differences in study design (randomized vs. non‐randomized clinical trials and non‐blinded trials vs. single‐ or double‐blinded trials), and variations in the clinical procedure performed (intracanal irrigation solutions, number of treatment visits, final restorative materials, type of teeth undergoing pulpectomy, and latency to follow‐up).

There are no reliable methods with which to quantify the amount of clinical, radiographical, and methodological heterogeneity. Careful selection of appropriate studies is the only way to ensure the derivation of accurate inferences in meta‐analyses. Despite attempts to include a large number of related studies in our analysis, our search yielded only 15 studies, two of which were deemed high‐quality studies suitable for inclusion. The small number of studies included in our meta‐analysis leading to substantial bias of heterogeneity (Von Hippel, 2015). To overcome this heterogeneity, we applied a random effects model and performed subgroup analysis; we feel that this allowed us to contrive reliable results.

## 5 CONCLUSION

On the basis of the current study findings, we believe that due to its resorbable property, Ca(OH)_2_/iodoform is the best filling material to be used for pulpectomy in primary teeth nearing exfoliation. Conversely, either ZOE or ZOE/iodoform combined with Ca(OH)_2_ is the materials of choice for pulpectomy in primary teeth need long time before exfoliation.

The clinical and radiographical success rates of Ca(OH)_2_/iodoform paste are comparable with that of ZOE in primary teeth pulpectomy up to ≥18‐month follow‐up.

Future clinical trials with a high‐quality randomized controlled clinical trials and long‐term follow‐up period are needed before a reliable conclusion can be drawn as to the best pulpectomy material in primary teeth.

## CONFLICT OF INTEREST

All authors declare no conflict of interest.

## Supporting information


**Figure S1.** Forest plot for meta‐analysis of the clinical and radiographic success rate of Ca (OH)2/iodoform compared to ZOE at 6 months follow up according to the type of irrigation
**Figure S2**. Forest plot for meta‐analysis of the clinical and radiographic success rate of Ca (OH)2/iodoform compared to ZOE at 12 months follow up according to the type of irrigation
**Figure S3**. Forest plot for meta‐analysis of the clinical and radiographic success rate of Ca (OH)2/iodoform compared to ZOE at ≥18 months follow up according to the type of irrigation
**Figure S4**. Forest plot for meta‐analysis of the clinical success rate of Ca (OH)2/iodoform compared to ZOE at 6 months follow up in relation to the type of teeth
**Figure S5.** Forest plot for meta‐analysis of the clinical success rate of Ca (OH)2/iodoform compared to ZOE at 12 months follow up in relation to the type of teeth
**Figure S6.** Forest plot for meta‐analysis of the clinical success rate of Ca (OH)2/iodoform compared to ZOE at ≥18 months follow up in relation to the type of teeth
**Figure S7.** Forest plot for meta‐analysis of the clinical success rate of Ca (OH)2/iodoform compared to; ZOE and ZOE/iodoform combined with Ca (OH) 2 at 6 months follow up according to study quality level (high and moderate quality)
**Figure S8**. Forest plot for meta‐analysis of the radiographic success rate of Ca (OH)2/iodoform compared to; ZOE and ZOE/iodoform combined with Ca (OH) 2 at 6 months follow up according to study quality level (high and moderate quality)
