# Supplementary material for: A comparison of calcium hydroxide/iodoform paste and zinc oxide eugenol as root filling materials for pulpectomy in primary teeth: A systematic review and meta‐analysis
Source: Clin Exp Dent Res. 2019 Mar 4;5(3):294–310. doi: 10.1002/cre2.173 (PMC6585588; doi:10.1002/cre2.173)
Supplement: Supplementary file 1 — Figure S1. Forest plot for meta‐analysis of the clinical and radiographic success rate of Ca (OH)2/iodoform compared to ZOE at 6 months follow up according to the type of irrigation Figure S2. Forest plot for meta‐analysis of the clinical and radiographic success rate of Ca (OH)2/iodoform compared to ZOE at 12 months follow up according to the type of irrigation Figure S3. Forest plot for meta‐analysis of the clinical and radiographic success rate of Ca (OH)2/iodoform compared to ZOE at ≥18 months follow up according to the type of irrigation Figure S4. Forest plot for meta‐analysis of the clinical success rate of Ca (OH)2/iodoform compared to ZOE at 6 months follow up in relation to the type of teeth Figure S5. Forest plot for meta‐analysis of the clinical success rate of Ca (OH)2/iodoform compared to ZOE at 12 months follow up in relation to the type of teeth Figure S6. Forest plot for meta‐analysis of the clinical success rate of Ca (OH)2/iodoform compared to ZOE at ≥18 months follow up in relation to the type of teeth Figure S7. Forest plot for meta‐analysis of the clinical success rate of Ca (OH)2/iodoform compared to; ZOE and ZOE/iodoform combined with Ca (OH) 2 at 6 months follow up according to study quality level (high and moderate quality) Figure S8. Forest plot for meta‐analysis of the radiographic success rate of Ca (OH)2/iodoform compared to; ZOE and ZOE/iodoform combined with Ca (OH) 2 at 6 months follow up according to study quality level (high and moderate quality) [file CRE2-5-294-s001.pdf]

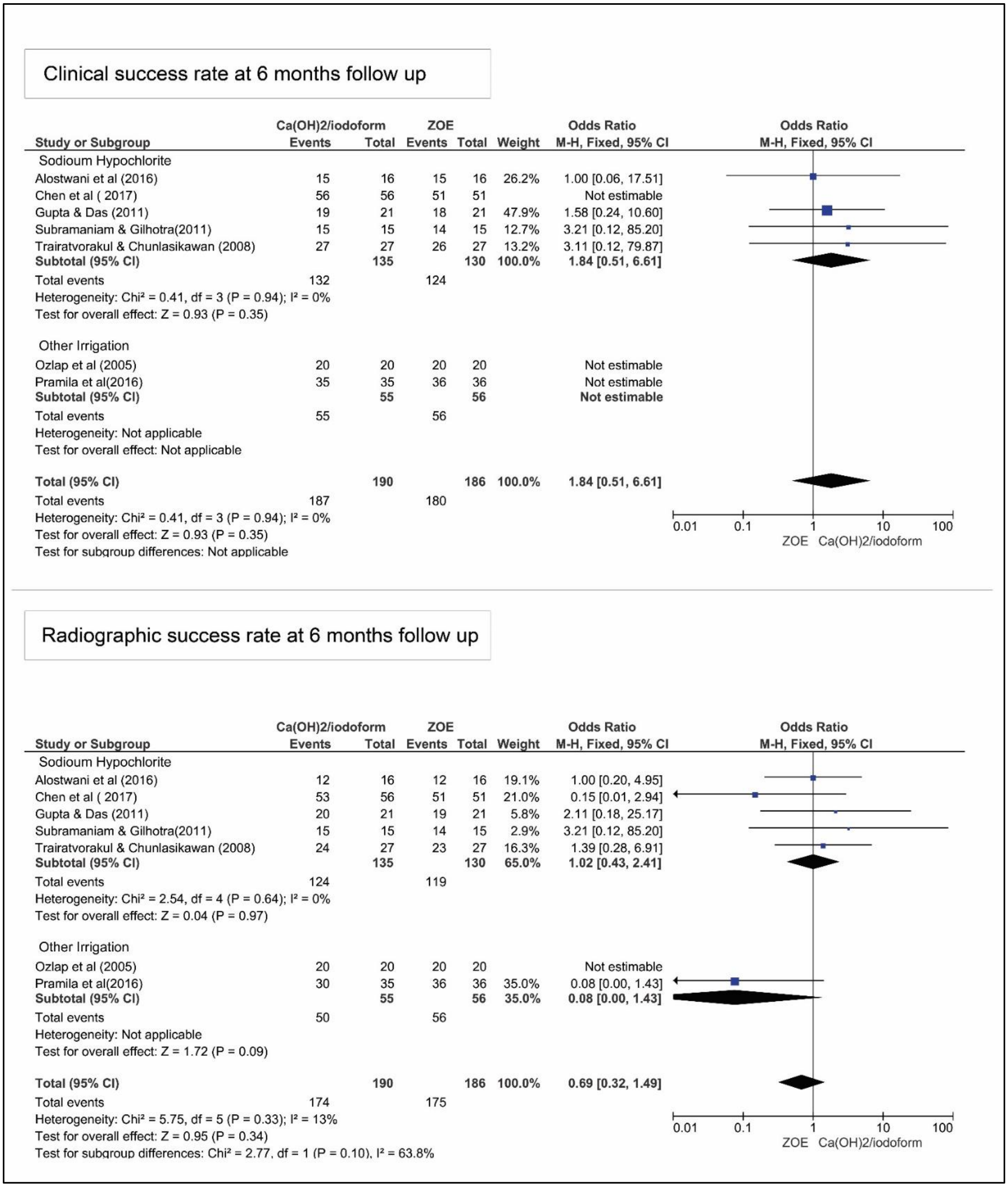

Figure S1. Forest plot for meta-analysis of the clinical and radiographic success rate of $Ca(OH)_2$/iodoform compared to ZOE at 6 months follow up according to the type of irrigation

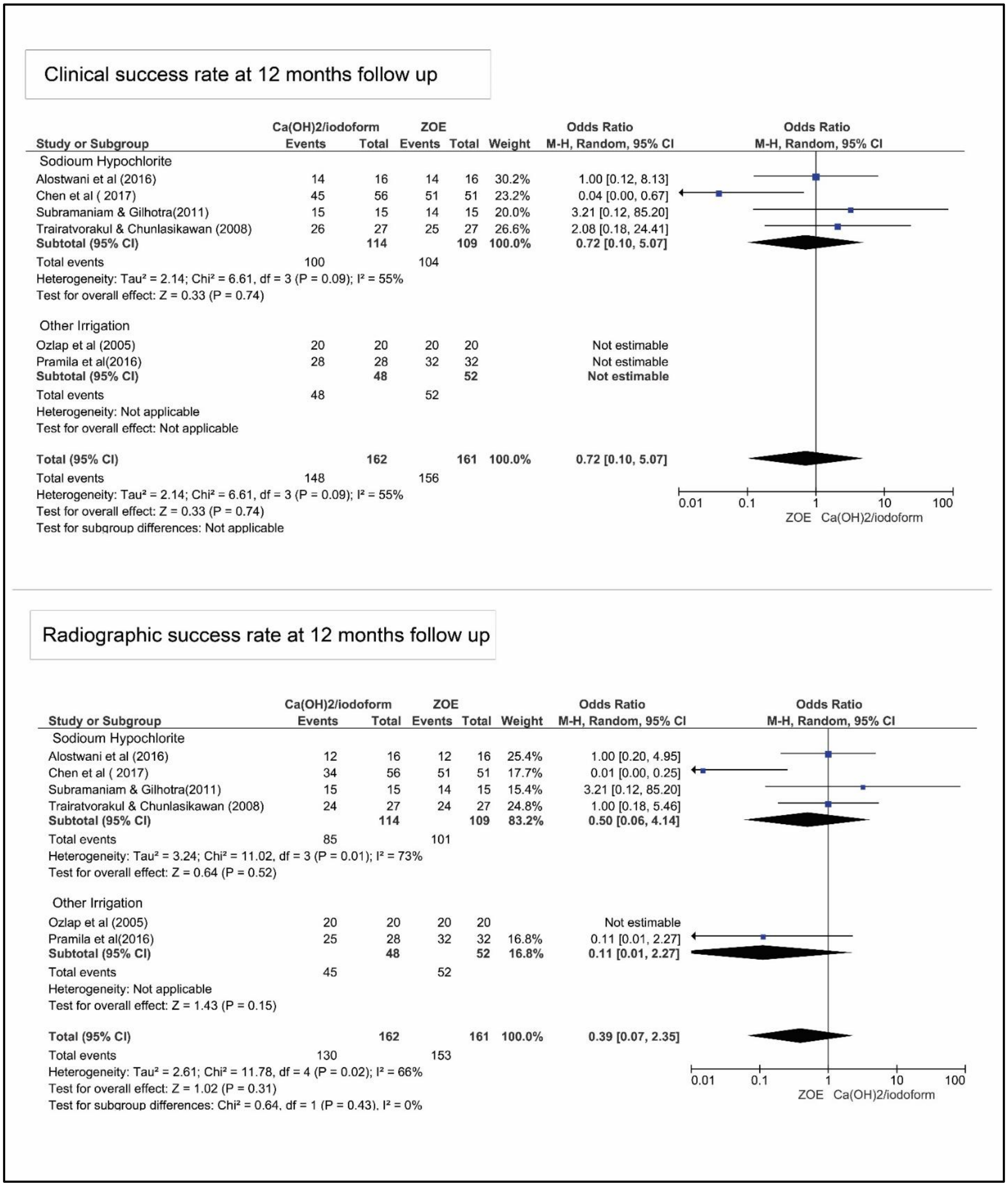

Figure S2. Forest plot for meta-analysis of the clinical and radiographic success rate of $Ca(OH)_2$/iodoform compared to ZOE at 12 months follow up according to the type of irrigation

Clinical success rate at ≥ 18 months follow up

Study or Subgroup	Ca(OH)2/iodoform Events	Ca(OH)2/iodoform Total	ZOE Events	ZOE Total	Weight	Odds Ratio M-H, Random, 95% CI
Sodioum Hypochlorite						
Subramaniam & Gilhotra(2011)	15	15	14	15	33.5%	3.21 [0.12, 85.20]
Chen et al (2017)	40	56	47	51	66.5%	0.21 [0.07, 0.69]
**Subtotal (95% CI)**		**71**		**66**	**100.0%**	**0.53 [0.04, 6.52]**
Total events	55		61			
Heterogeneity: Tau² = 2.12; Chi² = 2.34, df = 1 (P = 0.13); I² = 57%						
Test for overall effect: Z = 0.50 (P = 0.62)						
Other Irrigation						
Pramila et al(2016)	29	29	30	30		Not estimable
Ozlap et al (2005)	20	20	20	20		Not estimable
**Subtotal (95% CI)**		**49**		**50**		**Not estimable**
Total events	49		50			
Heterogeneity: Not applicable						
Test for overall effect: Not applicable						
**Total (95% CI)**		**120**		**116**	**100.0%**	**0.53 [0.04, 6.52]**
Total events	104		111			
Heterogeneity: Tau² = 2.12; Chi² = 2.34, df = 1 (P = 0.13); I² = 57%						
Test for overall effect: Z = 0.50 (P = 0.62)						
Test for subgroup differences: Not applicable						

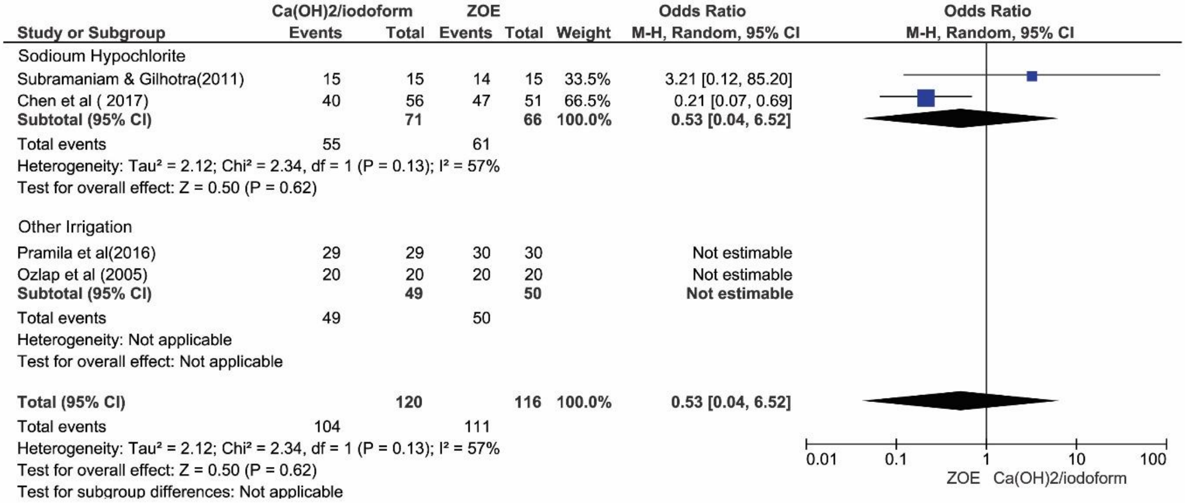

Radiographic success rate at ≥ 18 months follow

Study or Subgroup	Ca(OH)2/iodoform Events	Ca(OH)2/iodoform Total	ZOE Events	ZOE Total	Weight	Odds Ratio M-H, Random, 95% CI
Sodioum Hypochlorite						
Chen et al (2017)	30	56	45	51	61.7%	0.15 [0.06, 0.42]
Subramaniam & Gilhotra(2011)	15	15	14	15	19.0%	3.21 [0.12, 85.20]
**Subtotal (95% CI)**		**71**		**66**	**80.7%**	**0.46 [0.03, 8.23]**
Total events	45		59			
Heterogeneity: Tau² = 3.12; Chi² = 3.04, df = 1 (P = 0.08); I² = 67%						
Test for overall effect: Z = 0.52 (P = 0.60)						
Other Irrigation						
Ozlap et al (2005)	20	20	20	20		Not estimable
Pramila et al(2016)	28	29	30	30	19.3%	0.31 [0.01, 7.96]
**Subtotal (95% CI)**		**49**		**50**	**19.3%**	**0.31 [0.01, 7.96]**
Total events	48		50			
Heterogeneity: Not applicable						
Test for overall effect: Z = 0.71 (P = 0.48)						
**Total (95% CI)**		**120**		**116**	**100.0%**	**0.31 [0.06, 1.61]**
Total events	93		109			
Heterogeneity: Tau² = 0.87; Chi² = 3.10, df = 2 (P = 0.21); I² = 36%						
Test for overall effect: Z = 1.39 (P = 0.16)						
Test for subgroup differences: Chi² = 0.03, df = 1 (P = 0.86), I² = 0%						

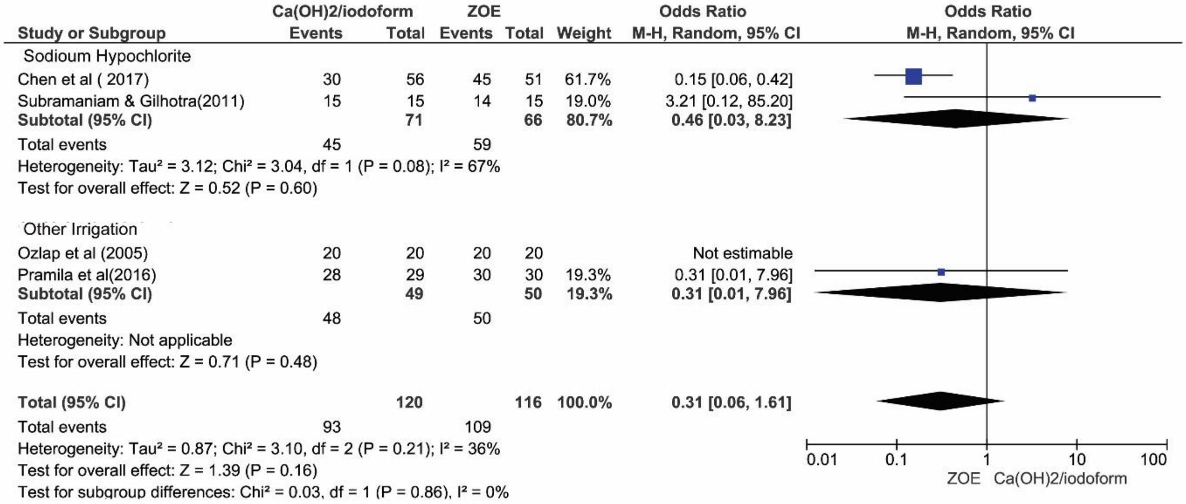

Figure S3. Forest plot for meta-analysis of the clinical and radiographic success rate of $Ca(OH)_2$/iodoform compared to ZOE at ≥ 18 months follow up according to the type of irrigation

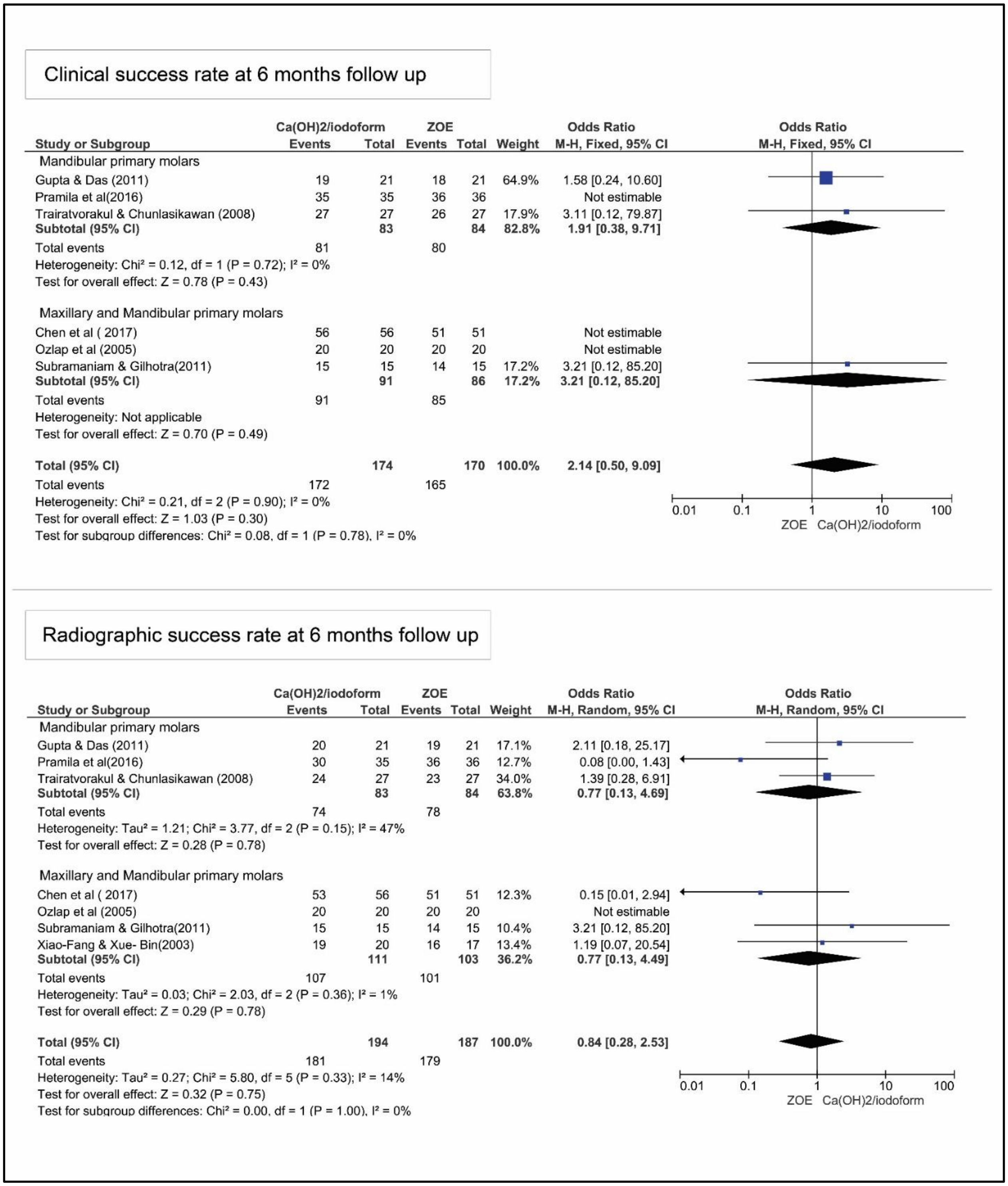

Figure S4. Forest plot for meta-analysis of the clinical success rate of $Ca(OH)_2$/iodoform compared to ZOE at 6 months follow up in relation to the type of teeth

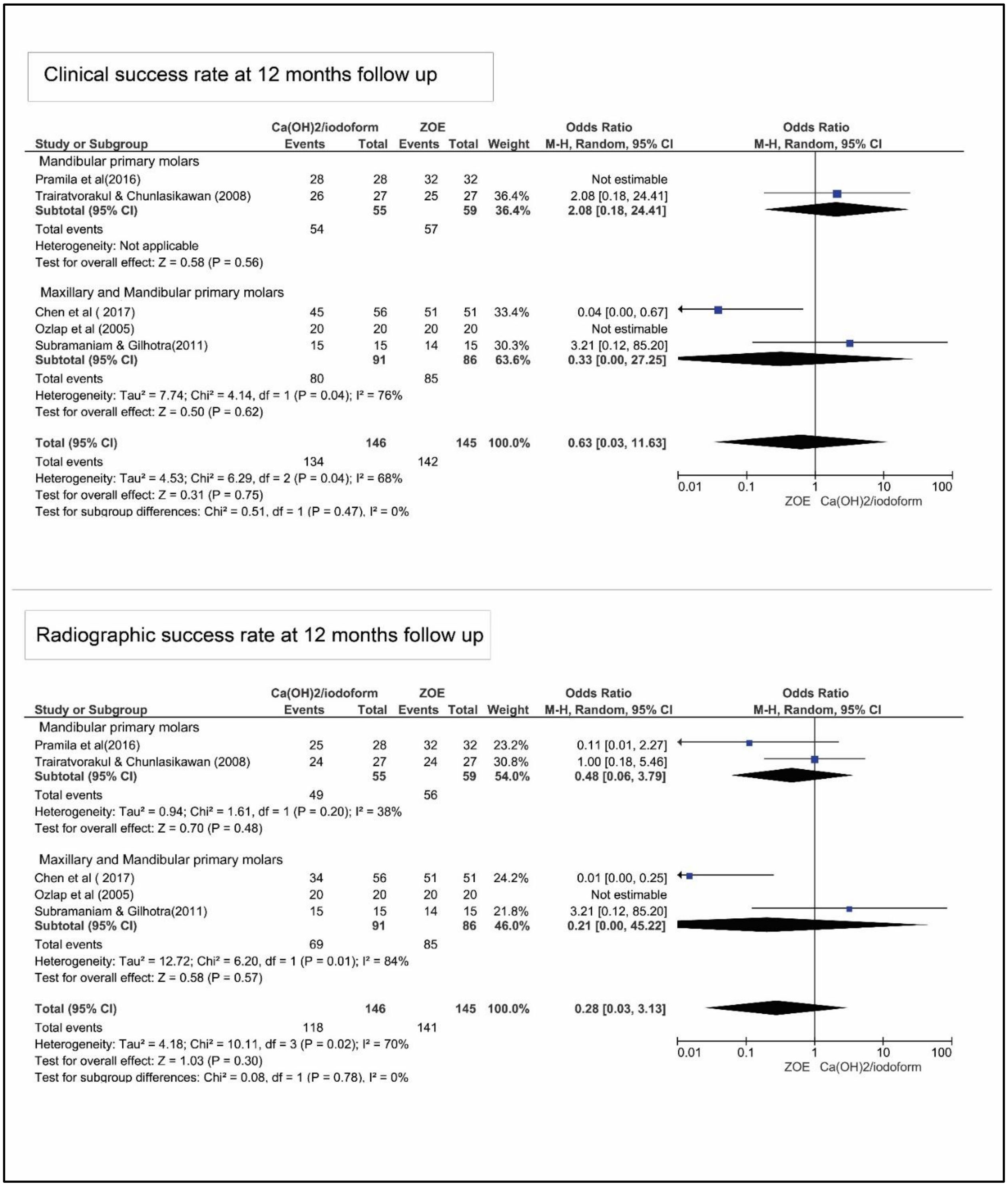

Figure S5. Forest plot for meta-analysis of the clinical success rate of $Ca(OH)_2$/iodoform compared to ZOE at 12 months follow up in relation to the type of teeth

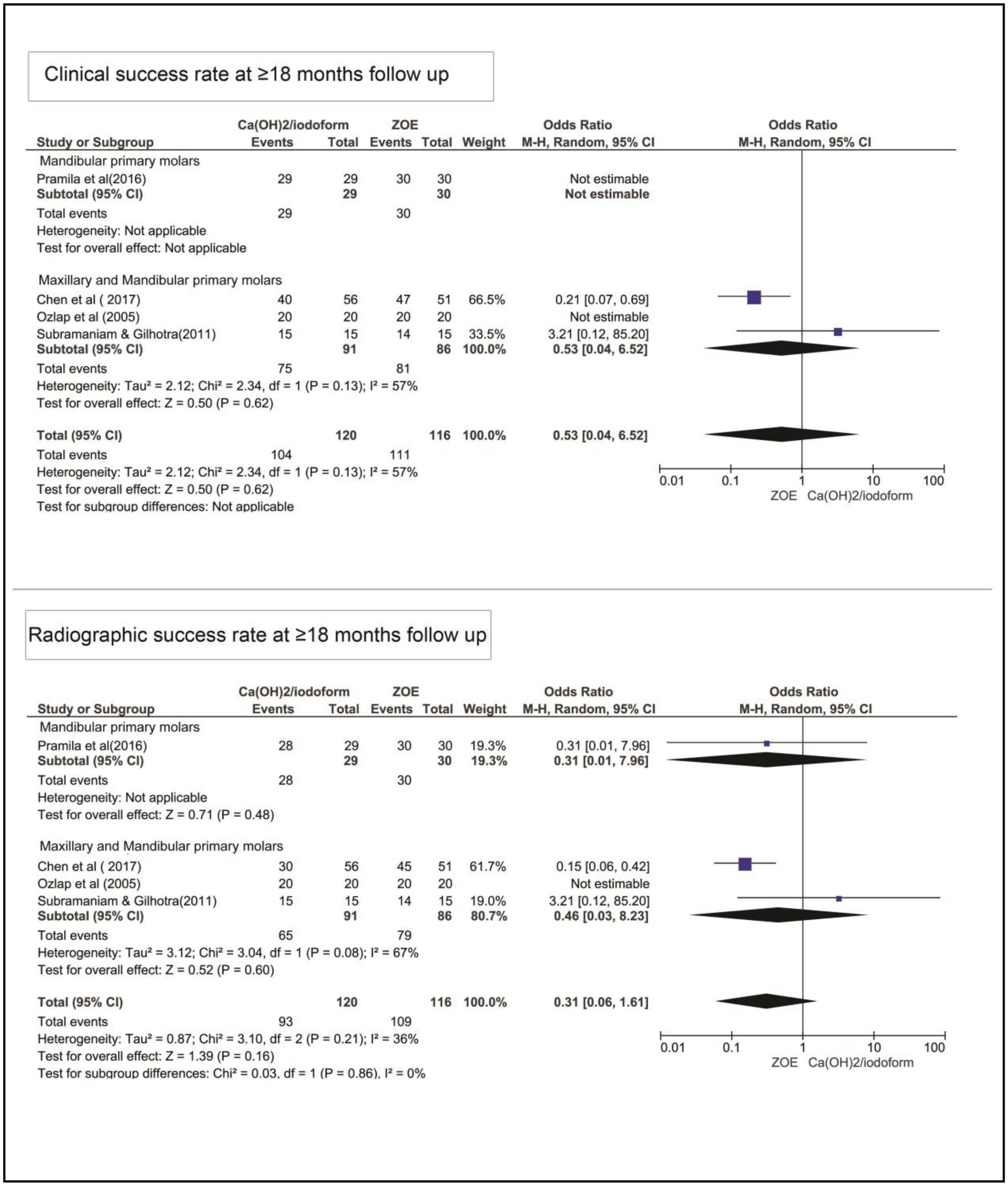

Figure S6. Forest plot for meta-analysis of the clinical success rate of $Ca(OH)_2$/iodoform compared to ZOE at ≥18 months follow up in relation to the type of teeth

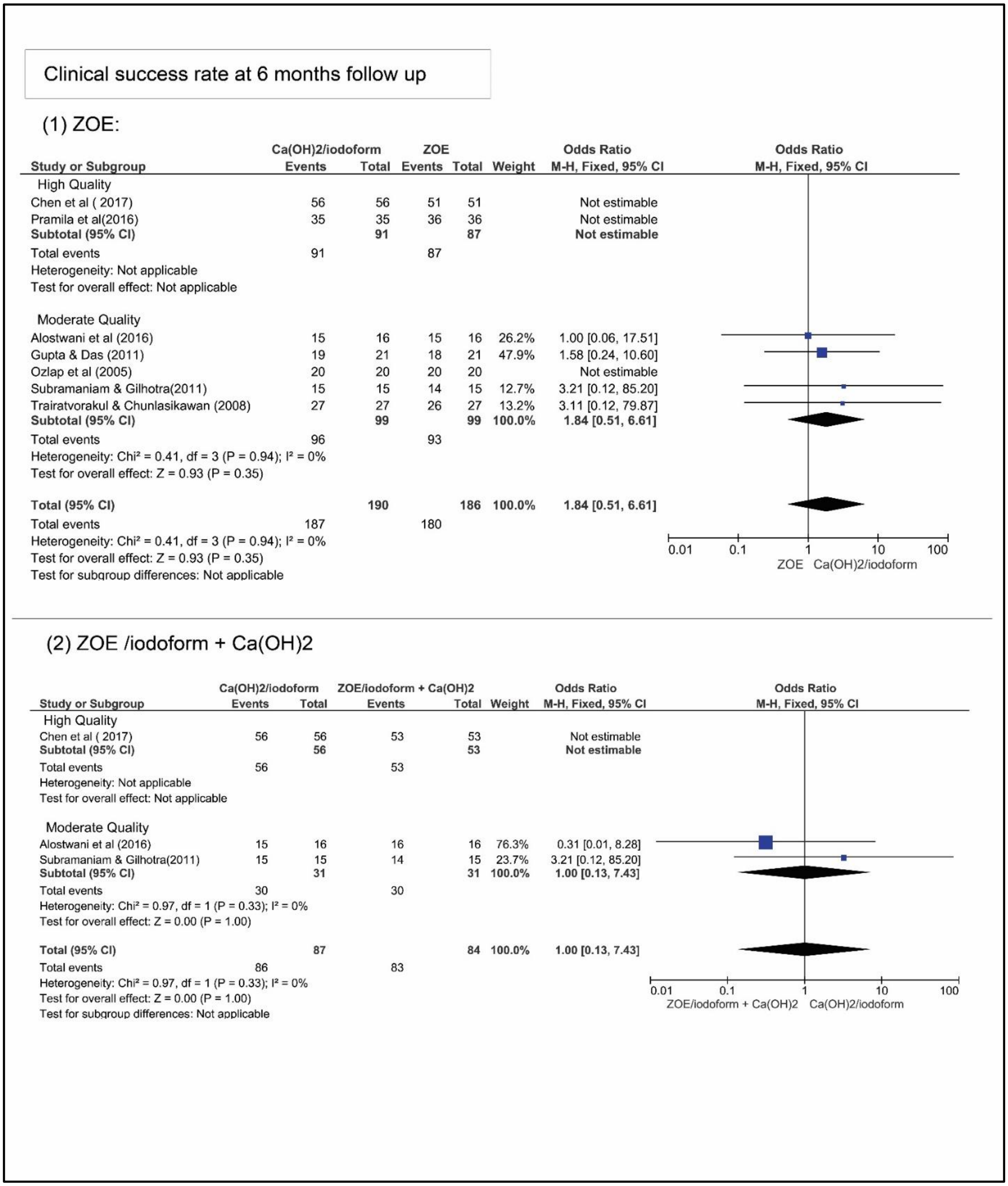

Figure S7. Forest plot for meta-analysis of the clinical success rate of $Ca(OH)_2$/iodoform compared to; ZOE and ZOE/iodoform combined with $Ca(OH)_2$ at 6 months follow up according to study quality level (high and moderate quality)

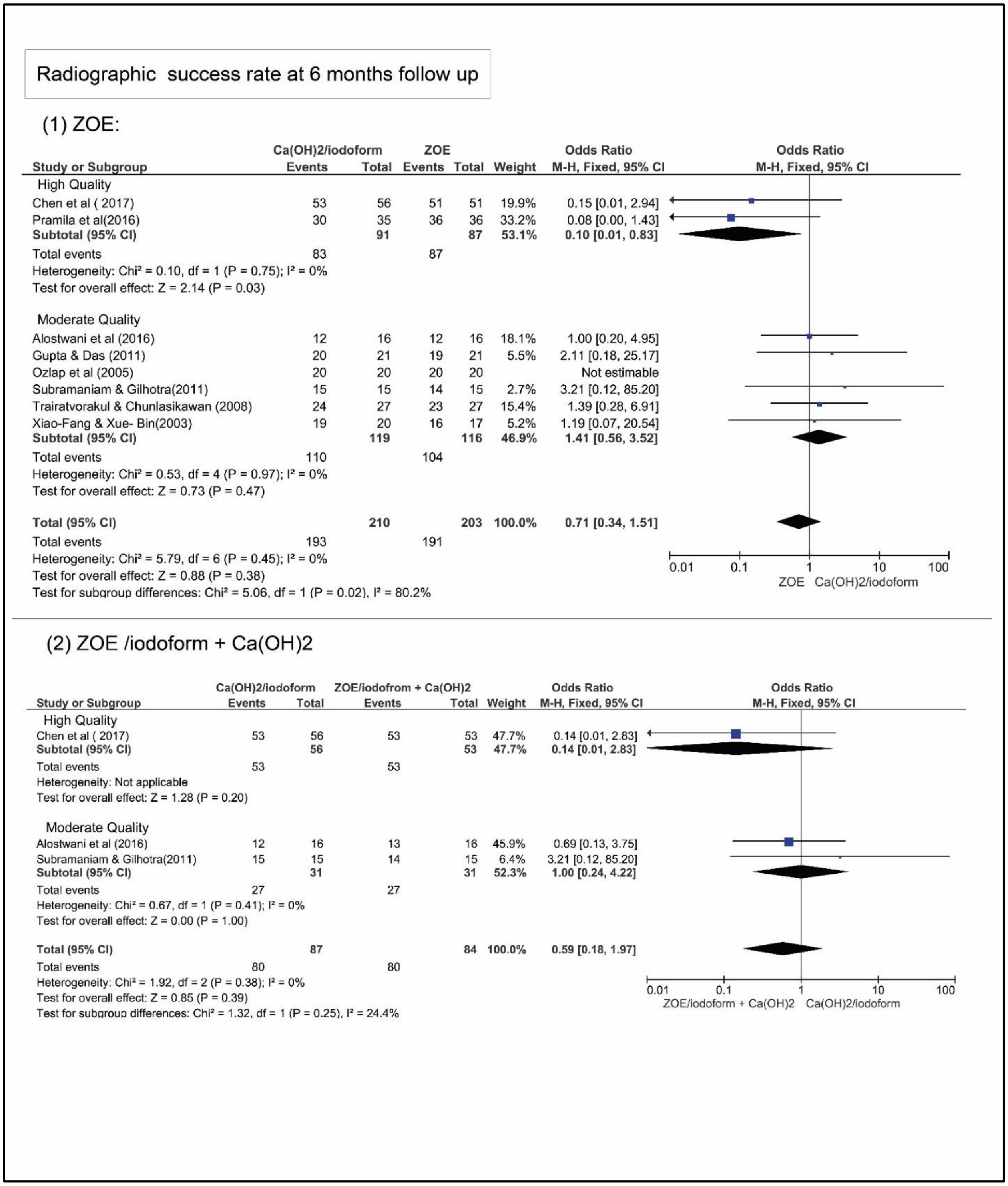

Figure S8. Forest plot for meta-analysis of the radiographic success rate of $Ca(OH)_2$/iodoform compared to; ZOE and ZOE/iodoform combined with $Ca(OH)_2$ at 6 months follow up according to study quality level (high and moderate quality)

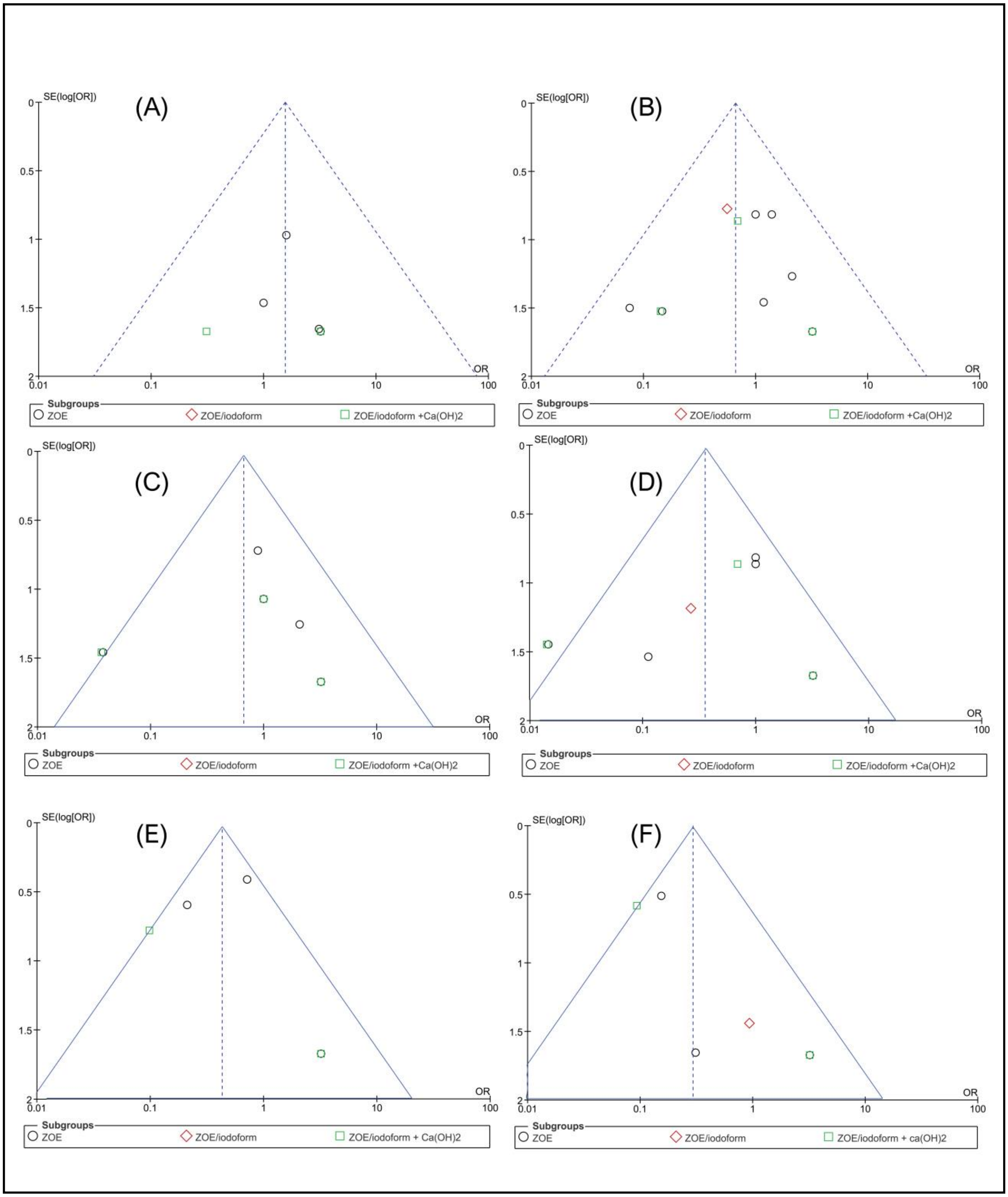

(A)
(B)
(C)
(D)
(E)
(F)
SE(log[OR])
OR
0.01
0.1
1
10
100
0
0.5
1
1.5
2
Subgroups
ZOE
ZOE/iodoform
ZOE/iodoform +Ca(OH)2
ZOE/iodoform + Ca(OH)2
ZOE/iodoform + ca(OH)2
